# Interplay among steroids, body condition and immunity in response to long-term captivity in toads

**DOI:** 10.1038/s41598-018-35495-0

**Published:** 2018-11-21

**Authors:** Stefanny Christie Monteiro Titon, Braz Titon Junior, Vania Regina Assis, Gabriela Sarti Kinker, Pedro Augusto Carlos Magno Fernandes, Fernando Ribeiro Gomes

**Affiliations:** Departamento de Fisiologia, Instituto de Biociências, Universidade de São Paulo, Sao Paulo, Brazil

## Abstract

Stressful experiences can promote harmful effects on physiology and fitness. However, stress-mediated hormonal and immune changes are complex and may be highly dependent on body condition. Here, we investigated captivity-associated stress effects, over 7, 30, 60, and 90 days on plasma corticosterone (CORT) and testosterone (T) levels, body index, and innate immunity (bacterial killing ability and phagocytosis of peritoneal cells) in toads (*Rhinella icterica*). Toads in captivity exhibited elevated CORT and decreased T and immunity, without changes in body index. The inter-relationships between these variables were additionally contrasted with those obtained previously for *R. schneideri*, a related species that exhibited extreme loss of body mass under the same captive conditions. While T and phagocytosis were positively associated in both species, the relationship between CORT and bacterial killing ability was dependent on body index alterations. While CORT and bacterial killing ability were positively associated for toads that maintained body index, CORT was negatively associated with body index in toads that lost body mass over time in captivity. In these same toads, body index was positively associated with bacterial killing ability. These results demonstrate that steroids-immunity inter-relationships arising from prolonged exposure to a stressor in toads are highly dependent on body condition.

## Introduction

Stress events and their intensity may be assessed by measuring glucocorticoid (GC) hormone levels in plasma and other fluids in most vertebrates^1^. While short-term stress response can be adaptive by promoting survival during fight-or-flight response, chronically elevated stress-associated GC levels may decrease fitness through several effects such as reproductive inhibition and depression of immune responsiveness^2–4^. During a stress response, elevated GC levels are associated with increased energy mobilization necessary to immediate and future needs^5^. Therefore, elevated GCs during long-term stress response may decrease individual’s body condition (e.g. mass relative to body length, body mass loss)^6–8^. Simultaneously, the reproductive axis is also influenced by stressors^9^. Studies on different vertebrates indicate that androgen plasma levels decrease in response to capture and confinement stress^10–13^. Moreover, stress-induced down-regulation of testosterone (T) plasma levels is more accentuated during chronic stress, when compared to acute stress^8,14,15^. Chronic stress still suppresses or imbalances immunity by decreasing proinflammatory cytokine production and suppressing mobilization and function of immune cells^4,16^.

The immunomodulatory role of GCs is well explored and described, especially for mammals, where bimodal effects depend on intensity and duration of exposure to stressors^4^. In this context, immunostimulatory parameters (e.g. cellular function and inflammatory responses) are frequently higher in response to acute elevation of GC levels, while suppressive immune effects (e.g. decreased immune cell proliferation and proinflammatory cytokine production) are more commonly observed under chronically elevated GC conditions^4,16^. In addition, a wide array of hormones, including androgens and leptin (a hormone that signalizes individual’s body condition), can modulate a vertebrate’s immune function^17,18^. Testosterone-induced immune suppression includes reduction of lymphoid tissues and decreased humoral and cellular immune responses^18,19^, while stimulatory effects are related to increased inflammatory events^20,21^. Furthermore, mounting an immune response requires a substantial energetic investment^22,23^. Accordingly, an animal with poor body condition, and consequent reduced endogenous energy availability, likely experiences suppressed immune function^24,25^. Thus, androgens, body condition and GCs can play an important and integrative role in the regulation of immune effectiveness^18^.

Experiments conducted on vertebrates in captivity have examined the relationships among plasma levels of GCs, sex steroids, body condition and immune function^19,26,27^. However, few studies have examined how captivity itself affects stress physiology and immune response, particularly in amphibians^8,28,29^. In this context, the first aim of the present study was to investigate the effects of captivity duration, specifically 7, 30, 60, and 90 days, on plasma corticosterone (CORT) and testosterone (T) levels, body index, and innate immune responses, measured as bacterial killing ability and phagocytosis of peritoneal cells, in male toads of *Rhinella icterica*. Given that previous studies suggested that long-term captivity (three months) is a chronic stressor for this species^29^, we tested the following hypotheses: 1) toads in captivity exhibit increased CORT and decreased T, body index and immune response when compared to wild ones (field values); 2) these effects are exacerbated throughout the time toads are kept in captivity; 3) CORT and immune response are negatively correlated over time in captivity; 4) T, body index and immune response are positively correlated over time in captivity. Additionally, *R. icterica* males were present in chorus during collection. Given that anuran calling behavior is associated with changes in CORT and androgens^30,31^, as well as to variation in innate immune response^32,33^, we compared males that were calling or not during the moment of capture. We expected that: 5) calling males show higher CORT and T than non-calling males under field conditions; 6) when brought to captivity, non-calling males show proportionally higher increase in CORT and calling males show proportionally higher decrease in T. Although there might be an influence of calling behavior in immunity, once all individuals would be maintained under the same conditions during long-term captivity maintenance, we additionally expected that 7) variation in immune responses over time in captivity is more associated with steroid plasma levels changes arising from captivity maintenance than with calling behavior at the moment of capture.

We have also observed that toads tend to respond differently to the same conditions of captive maintenance regarding body index variation. While adult male toads of *R. schneideri* displayed a marked body loss over time in captivity^8^, individuals of *R. icterica* in this study did not show variation in body mass while in captivity. Considering that the maintenance and activation of immune system is costly, and that the availability of energy resources is critical for an individuals’ survival^34,35^, the difference in body index in response to captive conditions might also contribute to a greater understanding of the associations between steroids, body condition and immunity in toads. Here, we analyzed the relationships among CORT, T, body index, and immunity in *R. icterica* and *R. schneideri* toads kept under the same captive conditions. Therefore, we tested the following additional hypothesis: 8) when body index decreases in response to long-term captivity, immune responses are directly associated with variation in body index and indirectly related with CORT and T; and 9) if body index does not vary over time in captivity, immune responses are directly associated with plasma CORT and T levels.

## Results

### Effects of captivity on body condition and physiological traits in *Rhinella icterica*

Descriptive statistics of variables from males of *R. icterica* in field and after captivity (7, 30, 60, and 90 days) are available in Supplementary materials (Tables S1 and S2). Body mass did not affect the physiological variables measured in this study either in field or in captivity (*P* ≥ 0.247; Tables S3 and S4).

**Figure 1:**
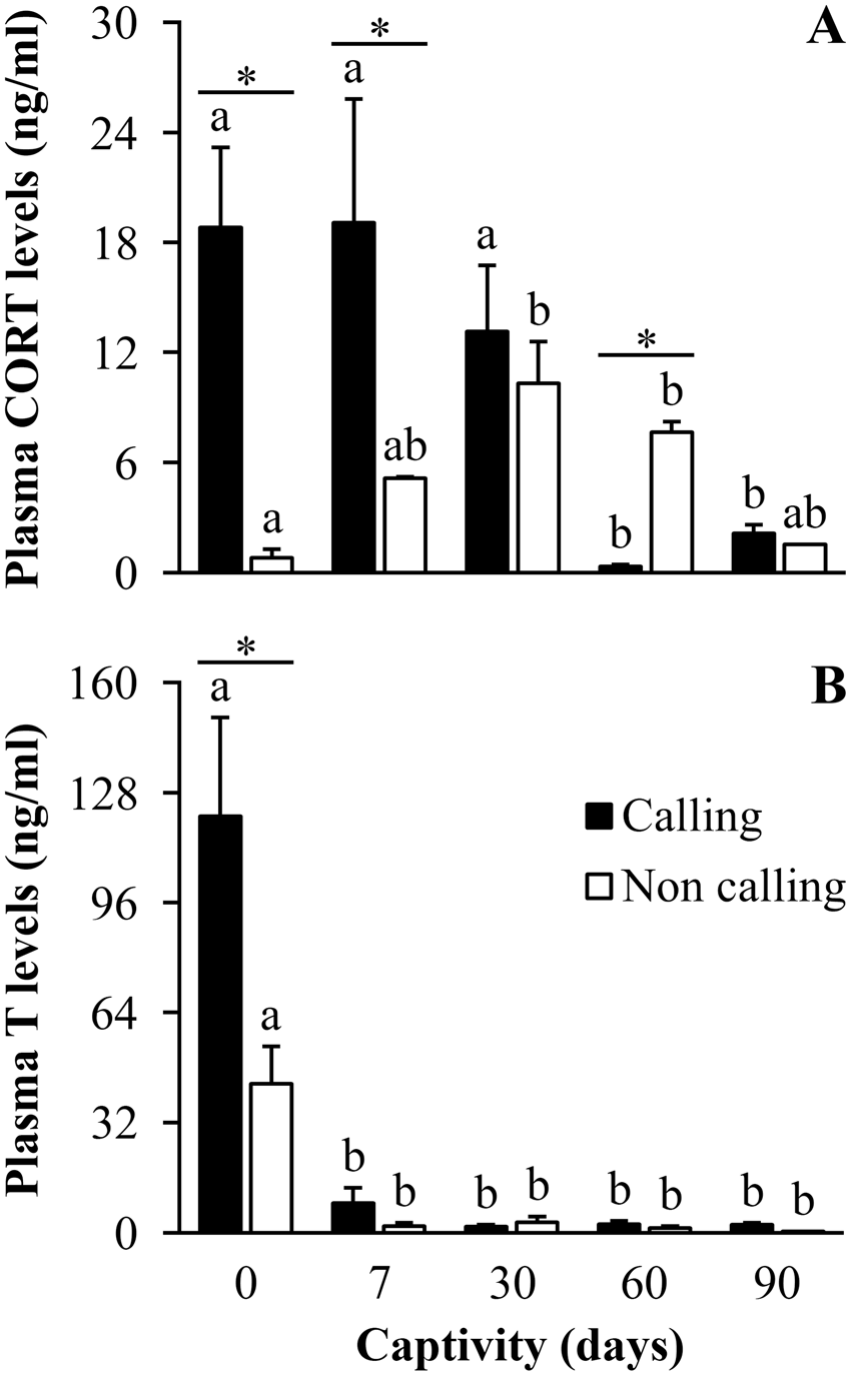
Field and captivity variation of plasma hormone levels of *Rhinella icterica* toads. Differences in (**A**) plasma corticosterone and (**B**) plasma testosterone levels of calling and non-calling individuals in the field and under captivity (*N* = 6 and 4 (0 - field), 4 and 2 (d7), 3 and 2 (d30), 3 and 3 (d60), 5 and 1 (d90) for calling and non-calling individuals, respectively, for both variables). Letters above the bars represent statistical differences for ANOVA, with different letters representing statistical difference within groups with *P* ≤ 0.05. Asterisks represent statistical differences between groups (calling or non-calling) at each specific time with *P* ≤ 0.05. Bars represent mean ± standard error. Abbreviations: CORT: Corticosterone; T: Testosterone.

Sixty four percent of the individuals captured in the field were engaged in calling activity, while the other toads were silent during the 10 min focal period. In the field, levels of CORT and T were higher in calling than non-calling toads (*P* ≤ 0.001; Fig. 1A and B), and the number of days in captivity affected CORT levels of calling and non-calling males in different ways (Fig. 1A, Table 1). Calling individuals showed sustained high CORT levels after 7 and 30 days in captivity, while non-calling animals exhibited a gradual increase in CORT levels over this period. There was no difference between callers and non-callers after 30 days (Fig. 1A, Table 1). Calling males showed an abrupt decrease in CORT levels after 60 and 90 days in captivity, while non-callers showed a more gradual decrease over the same period. After 60 days in captivity callers exhibited lower CORT levels than non-callers, and both groups showed equally low CORT levels after 90 days in captivity (Fig. 1A, Table 1). Although levels of T were higher for calling toads in the field, levels dropped sharply for callers and non-callers after just 7 days in captivity, and T was equally low for both groups throughout the period of captivity (Fig. 1B, Table 1).

**Table 1:** Effect of captivity duration and calling behavior on plasma steroid levels in *R. icterica* tested through a set of ANOVAs, with plasma corticosterone and testosterone levels as dependent variables and captivity duration (0, 7, 30, 60, and 90 days) and calling behavior (calling and non-calling) as factors.

Dependent Variable	Source	Type III SS	DF	MS	*F*	*P*
Plasma corticosterone levels	Corrected Model	128.846	9	14.316	9.766	<**0.001**
	Intercept	301.366	1	301.366	205.578	<**0.001**
	Calling behavior	7.296	1	7.296	4.977	**0.036**
	CD	30.468	4	7.617	5.196	**0.004**
	Calling behavior * CD	64.245	4	16.061	10.956	<**0.001**
	Error	33.717	23	1.466		
	Total	598.728	33			
	Corrected Total	162.563	32			
Plasma testosterone levels	Corrected Model	380.993	9	42.333	18.496	<**0.001**
	Intercept	212.222	1	212.222	92.724	<**0.001**
	Calling behavior	10.313	1	10.313	4.506	**0.045**
	CD	276.132	4	69.033	30.162	<**0.001**
	Calling behavior * CD	20.830	4	5.208	2.275	0.094
	Error	50.353	22	2.289		
	Total	811.290	32			
	Corrected Total	431.345	31			

Abbreviation as follow: Type III SS: Type III sum of squares; DF: Degrees of freedom; MS: Mean square; CD: Captivity duration. Variables with *P* significant < 0.05 are highlighted in bold.

**Figure 2:**
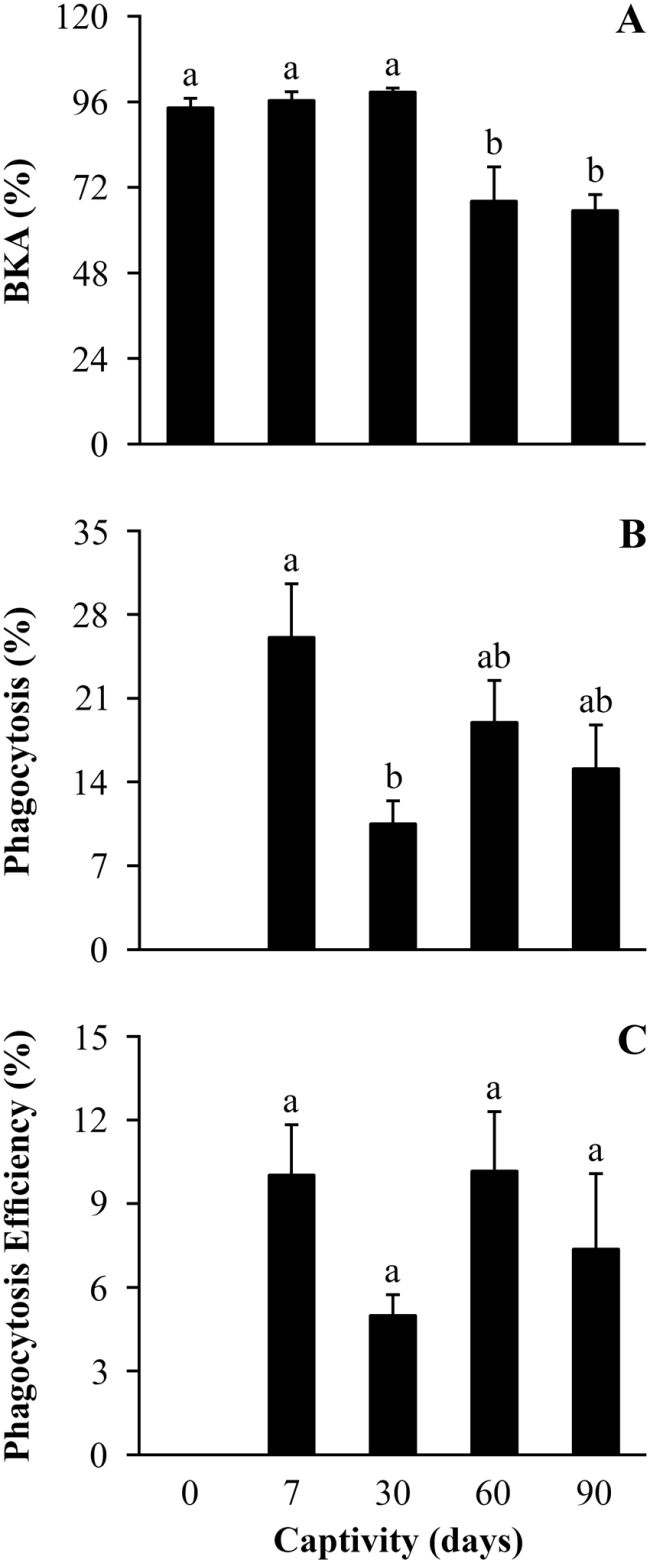
Field and captivity variation of immune response of *Rhinella icterica* toads. Differences in (**A**) Bacterial killing ability, (**B**) Phagocytosis (%) and (**C**) Phagocytosis efficiency (%) for individuals in field and captivity (*N* = 10 (0 - field), 6 (d7), 6 (d30), 6 (d60), 6 (d90) for all variables). Letters above the bars represent statistical differences for ANOVA, with different letters representing statistical difference with *P* ≤ 0.05. Bars represent mean ± standard error. Abbreviations: BKA: Bacterial killing ability.

**Figure 3:**
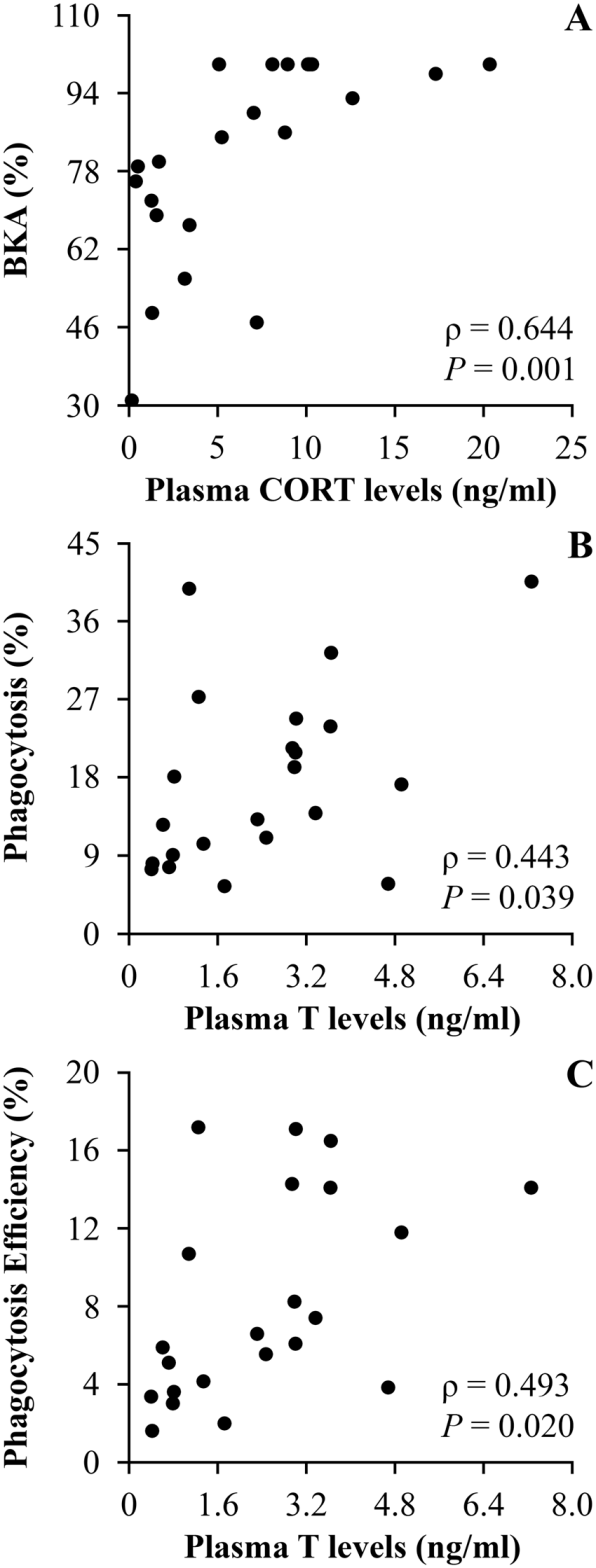
Correlations between plasma hormone levels and immune response in *Rhinella icterica* toads in captivity. (**A**) Correlation between CORT and BKA; (**B** and **C**) Correlations between T and phagocytic response. Abbreviations as follow: CORT: Corticosterone; BKA: Bacterial killing ability; T: Testosterone. (*N* = 22). Data were pooled to include all captivity durations (7, 30, 60, and 90 days in captivity).

Body index did not differ between callers and non-callers in the field, and remained steady while toads were in captivity (Table 2). There were also no differences in immune response measures between callers and non-callers in the field or throughout the period of captivity (*P* ≥ 0.145; Table S5). Bacterial killing ability decreased after 60 days in captivity and remained low by day 90 compared to values from the field and after 7 and 30 days in captivity (Fig. 2A, Table 2). Toads exhibited a transient reduction in phagocytosis percentage that was related to the duration of captivity, exhibiting the lowest values on the 30^th^ day in captivity (Fig. 2B, Table 2). Phagocytosis efficiency showed the same temporal trend of phagocytosis percentage in response to the duration of captivity, although this trend was non-significant (Fig. 2C, Table 2). Finally, levels of CORT were positively correlated with bacterial killing ability (Fig. 3A), and levels of T were positively correlated with phagocytosis percentage and efficiency (Fig. 3B and C) over time in captivity.

**Table 2:** Effect of captivity duration on body condition and immune response in *R. icterica* tested through a set of ANOVAs, with body index, bacterial killing ability, phagocytosis percentage and phagocytosis efficiency as dependent variables and captivity duration (0, 7, 30, 60, and 90 days) as factor.

Dependent Variable	Source	Type III SS	DF	MS	*F*	*P*
Body index	Corrected Model	99.430	3	33.143	0.200	0.894
	Intercept	1.953	1	1.953	0.012	0.915
	CD	99.430	3	33.143	0.200	0.894
	Error	2479.816	15	165.321		
	Total	2579.245	19			
	Corrected Total	2579.245	18			
Bacterial killing ability	Corrected Model	5879.014	4	1469.754	15.721	<**0.001**
	Intercept	166969.202	1	166969.202	1785.963	<**0.001**
	CD	5879.014	4	1469.754	15.721	<**0.001**
	Error	2617.712	28	93.490		
	Total	182957.828	33			
	Corrected Total	8496.727	32			
Phagocytosis	Corrected Model	780.847	3	260.282	3.499	**0.035**
	Intercept	7510.466	1	7510.466	100.973	<**0.001**
	CD	780.847	3	260.282	3.499	**0.035**
	Error	1487.625	20	74.381		
	Total	9778.938	24			
	Corrected Total	2268.471	23			
Phagocytosis efficiency	Corrected Model	109.301	3	36.434	1.552	0.232
	Intercept	1594.140	1	1594.140	67.915	<**0.001**
	CD	109.301	3	36.434	1.552	0.232
	Error	469.454	20	23.473		
	Total	2172.895	24			
	Corrected Total	578.755	23			

Abbreviation as follow: Type III SS: Type III sum of squares; DF: Degrees of freedom; MS: Mean square; CD: Captivity duration. Variables with *P* significant < 0.05 are highlighted in bold.

### Relationships among steroids, body condition and immunity in response to long-term captivity stress in *R. icterica* and *R. schneideri*

**Figure 4:**
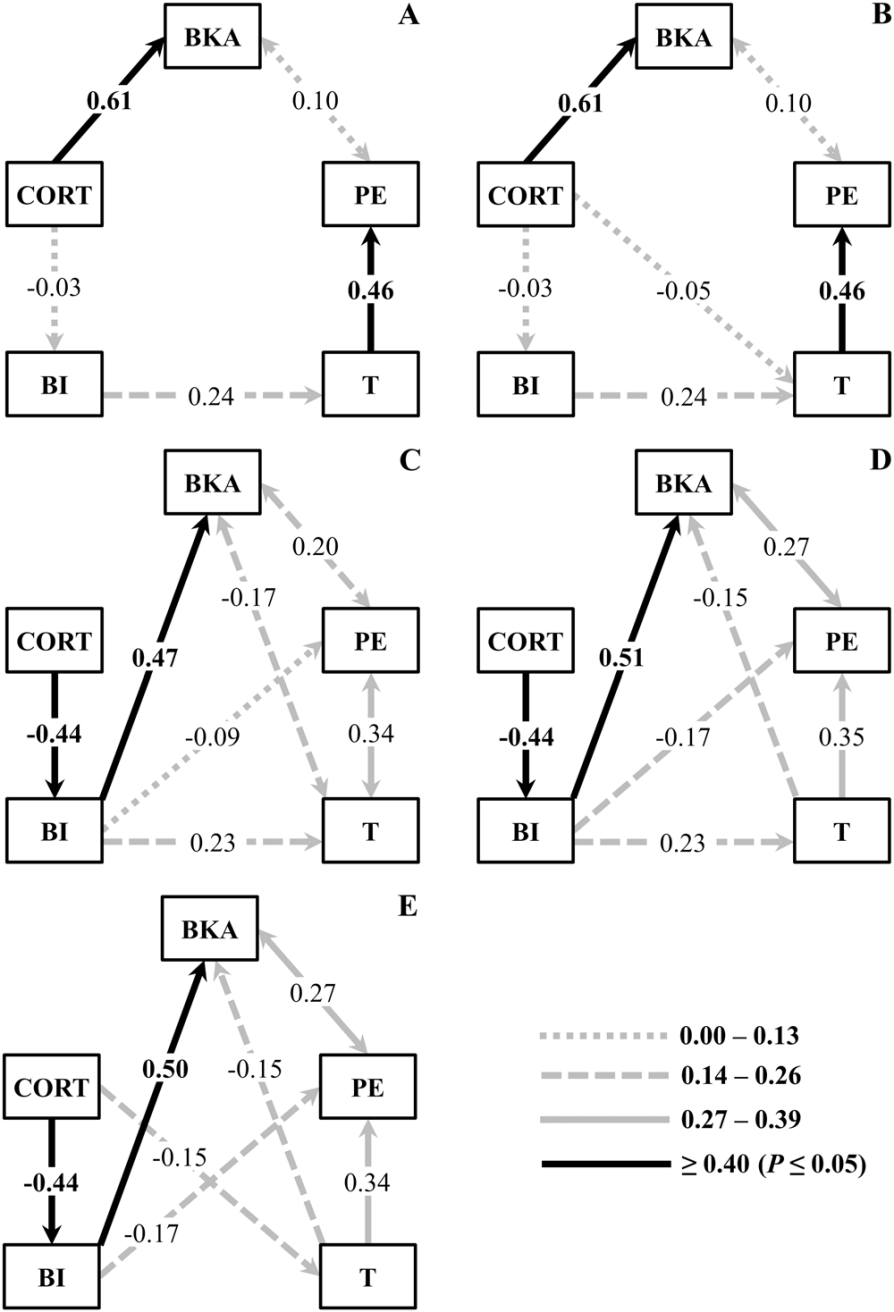
Selected structural equation modeling for body index, steroids and immune variables in *R. icterica* and *R. schneideri* in captivity. (**A** and **B**) Path diagrams of the two causal selected models for *R. icterica*. (**C**–**E**) Path diagrams of the three causal selected models for *R. schneideri*. Path coefficients shown are all standardized values in sequence with higher AIC and dAIC < 2.0: (A) Model 1 χ^2^ = 2.841, df = 5, P = 0.724, AIC = 610.9; (B) Model 7 χ^2^ = 2.782, df = 4, P = 0.595, AIC = 612.95; (C) Model 3 χ^2^ = 1.618, df = 3, P = 0.655, AIC = −97.1; (D) Model 4 χ^2^ = 1.618, df = 3, P = 0.655, AIC = −97.1; (E) Model 5 χ^2^ = 2.243, df = 3, P = 0.524, AIC = −96.4. Numbers within arrow indicate the completely standardized solution coefficient values. Positive numbers represent positive relations and negative numbers represent negative relations. One-arrow represents a regression result and two-arrow represent a correlation result. Abbreviations as follow: CORT: Plasma corticosterone levels; BI: Body index; T: Plasma testosterone levels; BKA: Bacterial killing ability; PE: Phagocytic efficiency. (*N* = 19 and 20, for *R. icterica* and *R. schneideri*, respectively).

The two models that best explain the relationships among CORT, T, body index, bacterial killing ability, and phagocytosis efficiency for *R. icterica* are depicted in Fig. 4A and B and on Table S6 (Supplementary Materials). Both models showed consistent results, reflecting the relationships among the investigated variables; there were positive influences of CORT levels on bacterial killing ability, and of levels of T on phagocytosis efficiency (Fig. 4A and B). A positive influence of body index on levels of T was also suggested for *R. icterica* during captivity in the models (Fig. 4A and B).

Three models emerged as those that best explained the relationships among the variables studied for *R. schneideri* (Fig. 4C–E; Table S6). All selected models showed consistent results regarding the relationships among CORT levels, body index, T levels, bacterial killing ability, and phagocytosis efficiency (Fig. 4C–E). Higher levels of CORT seem to negatively influence body index, which showed a prominent positive influence on bacterial killing ability and moderate effects on levels of T. T levels also positively influenced phagocytosis efficiency (Fig. 4C–E).

## Discussion

Our study demonstrated that, in addition to promoting complex time-dependent adjustments in CORT levels, toads held in captivity exhibited decreased levels of T and innate immune response in *R. icterica*. Trajectories of CORT levels for calling animals demonstrated that, at the moment of capture, calling toads exhibited higher CORT than non-calling. Calling individuals also maintained CORT levels similar to those in the field even after 7 and 30 days in captivity. Non-calling toads, in turn, exhibited increased levels of CORT while in captivity, with individuals at 30 days exhibited similar levels of CORT to calling ones. These findings are in line with our predictions given that calling behavior is directly associated with high CORT values^30,31^, and captive conditions result in higher CORT levels in anurans^8,27–29^. Interestingly, captive toads from both groups, calling and non-calling, experienced CORT levels similar to those of calling animals at the moment of the capture, suggesting that toads held captive for 30 days experience CORT levels at physiological levels of calling behavior in *R. icterica*. However, while transient increases in levels of CORT can contribute to the energy mobilization necessary for calling activity^36^, high levels of CORT over the long-term are indicative of chronic stress^1,4^.

Many vertebrates held in captive conditions exhibit increased levels of CORT^14,26,37^, including amphibians^28,29^. Nevertheless, whilst some anurans show sustained high levels of CORT^8,29^, others exhibit an attenuation of CORT levels over a period in captivity^27,28^. In our study, we observed that *R. icterica* toads exhibited sustained high levels of CORT for 30 days, but, contrary to our predictions, we observed pattern of decreased levels of CORT at days 60 and 90 when compared to values at 30 days in captivity. These results suggest that these animals can adjust to captive conditions, but only after a long period of captive maintenance. In the meantime, previous research describes sustained high levels of CORT for *R. icterica* after three months compared to baseline values^29^. Given that these toads were maintained under the same conditions, the contrast between our results and those reported previously^29^ may be attributed to the fact that animals were captured from different populations that were 500 km apart^38^, at different times of the year (July in this study and February in^29^)^39^, and in different years^40^.

Plasma T levels were higher in calling than non-calling toads in the field. Calling activity is often positively correlated with T in anurans^31,41^. While studies have shown that high levels of T are necessary to initiate and maintain calling activity in anurans^36,42^, some authors have also emphasized that exposure to a broadcast chorus stimulus can also increase levels of T^43,44^. In response to captivity duration, levels of T were much lower in toads after seven days in captivity for both calling and non-calling individuals. This result is consistent with the general pattern described for many vertebrates, including amphibians^12,45–47^. Levels of T decrease also in response to stressors such as inhibition of gonadotropin-releasing hormone secretion and impairment of the testicular function^12,48,49^. In this context, the decrease in levels of T in toads held long-term in captivity might be explained by the inhibition of the hypothalamic-pituitary-gonadal axis through the constant activation of the hypothalamic-pituitary-adrenal/interrenal axis^1,50^.

More importantly, we demonstrated that after the accentuated decrease in levels of T following the first days in captivity, T was sustained at similar low levels throughout captivity. In addition, as found previously for anurans^8,11,45,46^, there is no correlation between levels of CORT and T in *R. icterica*, suggesting that the decrease of T in response to captivity may not be directly mediated by changes in CORT. Stress-induced decrease in levels of T can occur at multiple physiological levels (e.g. inhibition of reproductive axis, acceleration of T clearance) and there may not be an associated change in CORT levels^1,36,51^. Further studies that focus on which T-mediated controlling mechanisms are influenced by stress response and GCs levels are necessary to better understand the stress T-induced changes in amphibians.

Despite the differences in baseline levels of CORT and T, and in CORT levels throughout a captive period, measures of immune responses were not different between calling and non-calling toads in the field or in response to captivity. We expected variation in the immune response associated with calling behavior because CORT and T are both modulators of the immune system^18,19^. However, all individuals of *R. icterica* were captured in a short period at the beginning of the breeding season, and body index, a common physiological condition associated to the immune response^24,25^, was not different between callers and non-callers. Because calling and non-calling toads were captured and held under similar conditions, and were subjected to the same captivity protocol, differences in immune responses between them might have been attenuated in the field and throughout the study.

As we predicted, pooled values for calling and non-calling animals showed that immune responses decrease following captivity in males of *R. icterica*, with the dynamics of suppressive effects varying according to the specific immune response variables. Compared to field conditions, toads showed a decreased plasma bacterial killing ability after 60 days in captivity, and this remained low through the 90^th^ day. In contrast, toads exhibited reduced phagocytosis percentage and phagocytosis efficiency after 30 days, followed by an increase in the 60^th^ in captivity. This is consistent with a previous report for captive male *R. icterica*, which exhibited decreased bacterial killing ability after 3 months^29^. Indeed, decreases in bacterial killing ability associated with stress, including long-term captivity, have been reported in birds and anurans^8,37,52,53^. Long-term stress conditions frequently suppress or deregulate immune responses by decreasing several cellular functions, including specific cytokine and antibody production, cell proliferation, and by inhibiting inflammatory processes (reviewed in^4^). Therefore, although the specific mechanisms remain to be investigated, the decreased bacterial killing ability following captive maintenance in *R. icterica* could be the result of reduced protein concentration, such as those from complement system.

Contrary to our prediction, bacterial killing ability was positively correlated with CORT levels throughout the captive period in *R. icterica*. Although CORT levels and immune responses are often negatively associated under chronic stress conditions, and after chronic treatment with exogenous corticosterone^4,54,55^, a positive association between CORT and immune responses has been described for amphibians in response to restraint stress and corticosterone treatment^53,56^. Therefore, our results are consistent with previous findings, and suggest that CORT may positively influence a toad’s immunocompetence under stress conditions. Interestingly, some studies showed decreased bacterial killing ability in response to stressors (short and long-term stress), but not correlated with increased CORT levels in anurans^8,53,57^, including *R. icterica* toads^29^. Therefore, although toads under stress conditions often exhibit a decreased bacterial killing ability, its immunosuppressive mechanisms may not rely on levels of CORT and its effects in all circumstances.

A transient decrease in the phagocytic activity, for both phagocytosis percentage and efficiency, was observed in *R. icterica* in response to captivity duration. This transient pattern in immune response is consistent with results of previous studies in birds and anurans^8,58^. Although animals experiencing stress may exhibit a decrease in many aspects of immunity, interspecific variation in immune response to stress is commonly observed, as well as intraspecific variation depending upon the immune parameter studied^8,37,53^. As we expected, phagocytosis percentage and efficiency were positively correlated with levels of T over time in captivity in *R. icterica*. Mostly known for its immunosuppressive role^18,19^, levels of T are positively correlated with immune response under stress conditions in birds and anurans^8,47^. These findings suggest that high levels of T may enhance some aspects of the activity of immune cells. Nevertheless, more studies associating T manipulation and immune response *in vivo*, as well as *in vitro*, are necessary to increase our understanding of the effect of T on the immune system of toads.

Regarding the relationships among steroids, body condition and immunity in *Rhinella* toads, our results suggest that plasma levels of T and CORT may positively influence features of innate immunity, measured as phagocytosis efficiency and bacterial killing ability, in toads that exhibit better body condition. Interestingly, plasma T levels showed a stimulatory effect associated with cellular aspects of immunity (phagocytic activity) for both conditions (presence or absence of body index variation in response to long-term captivity stress). Besides its general immunosuppressive effects, a meta-analysis showed that T might enhance cell-mediated immune responses in many vertebrates^59^. Studies of the effects of T on immune cells show that neutrophil activity and cytokine production by CD4^+^ lymphocytes are enhanced by T treatment^60,61^. Moreover, effects of T may interact with the effects of body condition for mounting and maintaining immune responses. In this way, individuals in better body condition concomitantly with higher levels of T can afford a better immune response^21,62^. Our results are in accordance with those aforementioned, since toads with higher levels of T showed a tendency to exhibit a better BI and the highest values for phagocytosis efficiency throughout time in captivity. Nevertheless, more studies with dietary controlling conditions are important to highlight the energetic state role in modulating the interactions between T and immunity in toads.

For those toads not showing variation in body condition in response to long-term captivity, our results showed that CORT is positively associated with bacterial killing ability. Plasma bacterial killing ability reflects the activity of soluble proteins, such as complement proteins, natural antibodies, and lysozymes in response to foreign microorganisms^63^. Accordingly, during long-term stress condition, chronic stress response leads to a decrease in natural antibodies and levels of complement proteins and, in turn, a reduction in plasma innate immunity (reviewed in^4^). Our results are consistent with the well-documented long-term stress-induced suppressive effects on immune responses. However, we found a positive correlation between CORT and bacterial killing ability in animals presenting no variation in body index in response to chronic stress, suggesting that bacterial killing ability can be positively modulated by CORT in toads capable of maintaining good body condition over long-term stressful conditions. Increased humoral immunity (antibody titers) and a trend to increase bacterial killing ability in response to repeated elevation of corticosterone (transdermal application) has being previously described in lizards^64^. Additionally, there are some studies showing that treatment with GCs (at baseline and stress-induced concentrations) can promote overexpression of cytokines (tumor necrosis factor, for example) and also immune-related transcriptional factors (nuclear factor kappa B, for example), in mammal immune cells^65,66^, pointing to a GC-induced immune-enhancing role. Particularly in anurans, CORT transdermal application increased blood phagocytic ability but showed no effects on bacterial killing ability^46^. Further research on CORT treatment is necessary to assess how, and in which contexts CORT levels can directly influence toad’s immune system.

In toads that exhibited a decrease in body index over time in captivity, individuals showing higher CORT were characterized by lower body index and, consequently, by lower bacterial killing ability. These results indicate that body condition is positively associated with this aspect of immunity in toads. Increased levels of CORT stimulate glycogenolysis, lipolysis, and facilitate the breakdown of stored triglycerides^3,67^. Therefore, high levels of CORT can negatively influence body condition by decreasing energetic reserves under chronic stress conditions^67^. Likewise, decreased body index associated with reduced bacterial killing ability might be a result of the sustained high CORT levels, leading to greater loss of body mass over time in captivity in some toads^8^. The innate immune system is the first line of defense against pathogens for most vertebrates, and its maintenance at baseline levels is necessary for constant surveillance^68^. Given that immune responses may be energetically expensive^23^, individuals in better body condition may exhibit better immunity. Moreover, reduced total body fat, an indicator of body condition, correlates with impaired immunity in a wide range of species (reviewed in^22^). In mammals, surgical removal of adipose tissue impairs antibody production, with immune function being restored after compensatory regrowth of fat pads^69^. Moreover, body condition can be sensed by immune cells through plasma leptin levels^17,34^. In a study conducted by Demas and Sakaria^17^, lipectomy decreased circulating leptin and humoral immunity, whereas restoring leptin via treatment with exogenous leptin restored lipectomy-induced immune suppression. Therefore, it is possible that individuals in poor body condition display a reduced immunity in response to low levels of leptin throughout captivity. In this way, the results from SEM analyses suggest that CORT, body index, T and immune responses can be directly associated in anurans. Studies with controlled diet combined with exogenous steroid application, using individuals from one species^70^, would be an interesting avenue of experimental tests for these suggested causal relations.

## Conclusions

Captivity maintenance resulted in high levels of CORT and decreased levels of T in *R. icterica* over a prolonged period of captivity. Immune response may concurrently vary over time in captivity, and depends on the immune parameter studied. While toads exhibited a transient decrease in phagocytosis efficiency, they also exhibited a consistent decrease in bacterial killing ability in response to long-term captivity. Additionally, bacterial killing ability is positively correlated with levels of CORT, and phagocytosis efficiency is positively associated with levels T during captivity. These results suggest that captive maintenance can be considered a stressor for *R. icterica*, and is associated with multiple hormone-immune interactions.

Additionally, the analyses of endocrine-immune responses of toads (*R. icterica* and *R. schneideri*) under the same captive conditions reveal patterns of common covariance and functional implications. While levels of T and phagocytosis showed a consistent and similar positive relationship throughout period of captivity, the relationship between levels of CORT and bacterial killing ability depended on a toad’s body index. Levels of CORT and bacterial killing ability are positively associated in toads that maintain body index throughout the period under captivity. Otherwise, CORT is negatively associated with body index, which is positively associated with bacterial killing ability, in those animals characterized by lowering body index in response to captivity duration. Furthermore, a better body index tends to be associated with higher levels of T. Collectively results of this study indicate coordinated changes in steroid plasma levels (CORT and T) and different immune parameters over time in captivity. Moreover, body condition may play a critical role in modulating the interactions among CORT, T and immune responses in toads. Because resources in nature may vary according with environmental conditions and can result in possible energetic trade-offs, our results suggest that toads in a long-term stress condition may experience reduced immunity, with individuals in poorer body condition being more susceptible to impairment of the immune response.

## Material and Methods

### Animals and study site

*Rhinella icterica* and *R. schneideri* are both species belonging to the *Rhinella marina* group, characterized by large body size and broad geographical distribution^71^. *Rhinella schneideri* occurs predominantly in the Cerrado, but is also present in the Atlantic Forest, Amazon Forest and Caatinga^71^; the geographical distribution of *R. icterica* is restricted more to Atlantic Forest, but populations also occur in some areas of Cerrado^71^.

Thirty-eight adult males of *R. icterica* were collected in Botucatu (22° 53′ 11.8″S, 48° 29′ 23.2″W) - São Paulo/Brazil in July 2015. Animals were collected under license from Instituto Chico Mendes de Conservação da Biodiversidade (ICMBio, process number 8132-1). All experiments and procedures were approved by IB-USP Ethical Committee (CEUA - n° 054/2013) and performed in accordance with relevant guidelines and regulations of the Brazilian law for scientific use of animals (Federal Law – n° 11.794/2008). Data related to field collection and captivity maintenance for *R. schneideri* were obtained from Titon *et al*.^8^.

### Capture and blood sample collection

*Rhinella icterica* toads (N = 10) were located by visual inspection and captured by hand. Blood samples (~200 µl) from those males were collected in the field (July 2015) by cardiac puncture using heparinized 1 ml syringes and 26 Gx1/2” needles. Only samples collected within 3 min after animal disturbance were considered in order to avoid influence of handling on hormone levels^72^. After blood collection, these animals were weighed (0.01 g) and the snout-vent length using a digital caliper (0.01 mm) was measured. These ten toads were kept isolated to avoid resampling, but eventually released in the field at the end of field work. Blood samples were labeled and kept on ice (<4 hours) until they were centrifuged to isolate the plasma (4 min at 604 g). Plasma samples were stored in cryogenic tubes, and kept in liquid nitrogen until they could be transferred to a −80 °C freezer, for hormone assays and bacterial killing ability. Another twenty-eight males were captured (not bled in field), transported to the laboratory and kept in captivity (see captive conditions, and duration and experimental design sections for further information). The captive individuals (N = 28) were weighed and kept in individual plastic containers, covered by lids with holes to allow air circulation, with free access to water for three days until they were taken to the laboratory. During this time the animals were exposed to the natural local climate and photoperiod conditions.

All *R. icterica* toads were found in an active chorus at the moment of capture. Since *R. icterica* males call on the shore of the pond^73^, once we identified the chorus, we approximated the core of the pond in order to identify the individual performing calling behavior. After identifying the toads, we observed them for 10 minutes. Individuals that presented calling behavior within this interval were considered callers^30^. Individuals that did not present calling behavior within 10 minutes and were observed far from the pond for at least 2 m were considered non-callers^30,73^. The presence or absence of calling behavior (calling [N = 23] or non-calling [N = 12]) at the moment of capture was recorded for each individual (those released in field and the ones kept in captivity) and included in further analysis.

### Captive conditions

At the laboratory, toads were kept in captivity under the same conditions of *R. schneideri* in Titon *et al*.^8^. The animals were individually housed in plastic containers (43.0 cm × 28.5 cm × 26.5 cm). The lids of the containers had holes to allow air circulation. Toads were exposed to an 11/13 LD cycle (lights on at 7:40 am and off at 6:40 pm) and temperature of 21 ± 2 °C, based on its preferred temperature, 22 °C^74^. The animals had free access to water, and were fed cockroaches once per week. Toads were weighed two days before the experimental procedure. Captive conditions were the same for all individuals, with captivity duration varying among experimental groups, as described below.

### Captivity duration and experimental design

Toads were divided in four groups to be sampled after 7, 30, 60, and 90 days in captivity to allow us to evaluate the effects of captivity duration on levels of CORT and T, and immune response (bacterial killing ability and phagocytosis of peritoneal cells). A blood sample from each individual was collected and processed according to the methods described above. Plasma samples were used for bacterial killing ability and hormone assays. After blood collection, animals were euthanized by immersion in a lethal solution of benzocaine (0.2%), the snout-vent length was measured, and then the retrieval of peritoneal cells was performed. Blood collection and retrieval of peritoneal cells were performed between 19:00 and 20:00.

### Phagocytosis

Once the animals were euthanized, the lavage fluid (cells + PBS) of the peritoneal cavity was collected with sterile surgical material according to Titon *et al*.^8^. Lavage fluid was centrifuged (259 g, at 4 °C for 9 min), the supernatant was discarded, and cells were resuspended in 1 ml of PBS to perform phagocytosis assay. Due to methodological limitations in field, this assay was carried out only for individuals in captivity (7, 30, 60, and 90 days).

The zymosan phagocytosis assay of peritoneal cells of *R. icterica* was carried out following a protocol^8^. Briefly, aliquots of 200 µl of the lavage fluid (PBS with macrophages and neutrophils adjusted to 1 × 10^6^ cells/ml) were diluted in 800 µl of PBS. Subsequently, 100 µl of zymosan (SIGMA Z-4250 A-Carboxyfluorescein Diacetate Succinimidyl Ester, at a concentration of 1 × 10^7^ particles/ml PBS) were added to the samples, followed by 35 min of incubation at 25 °C. A negative control was made with the lavage fluid diluted in PBS in the same proportion. Reactions were stopped by adding 2 ml of EDTA solution (6 mM). After centrifugation (259 *g*, at 4 °C for 7 min), the supernatant was discarded and 200 µl of paraformaldehyde (1%) were added. The samples were then kept at 4 °C for 1 hour for cell fixation. Thereafter, 500 µl of PBS were added and the samples were centrifuged (259 g, at 4 °C for 7 min). Supernatant was discarded and 100 µl of sterile PBS were added for flow cytometry.

Samples were analyzed on a Flowsight imaging flow cytometer (Merck-Millipore, German) interfaced with a DELL computer. Data from 20,000 events were acquired utilizing the 488 nm laser at a 20x magnification, through INSPIRE software. Macrophages and neutrophils were identified through gate images in focused-single cells plotted on the bright field area *vs*. the side scatter plot. Quantification of phagocytosis was estimated by mean zymosan-carboxyfluorescein diacetate succinimidyl ester fluorescence cell. The percentage of cells that engulfed at least one zymosan particle (with green fluorescence divided by the total number of cells [multiplied by 100]) was expressed as the phagocytosis percentage. The percentage of cells that ingested three or more zymosan particles was expressed as phagocytosis efficiency. Acquired data were analyzed using IDEAS analysis software (EMD Millipore) version 6.1 for windows.

### Bacterial killing ability

The plasma bacterial killing ability of *R. icterica* was assessed by the assay conducted according Assis *et al*.^75^. Briefly, 10 µl of plasma was combined with 10 µl of bacteria (*Escherichia coli* diluted to 10^6^ microorganisms per ml) and 190 µl of Ringer’s solution. Positive controls consisted of 10 µl of bacteria in 200 µl of Ringer’s solution, and negative control contained 210 µl of Ringer’s solution. All samples and controls were incubated by 60 min at 37 °C (optimal temperature for bacterial growth). Tryptic soy broth (500 µl) was then added to each sample. The bacterial suspensions were thoroughly mixed and 300 µl of each one was transferred (in duplicates) to a 96-well microplate. The microplate was incubated at 37 °C for 2 h, and thereafter the optical density of the samples was measured hourly in a plate spectrophotometer (wavelength 600 nm). The bacterial killing ability was evaluated at the beginning of the bacterial exponential growth phase, and calculated according to the formula: *1 - (optical density of sample/optical density of positive control)*, which represents the proportion of killed microorganisms in the samples compared to the positive control.

### Hormonal assays

A single ether extraction was performed on 10 µL of plasma^8^, and CORT and T concentrations were then determined in duplicate in standard ELISA kits (CORT number 501320; T number 582701, Cayman Chemical), according to the manufacturer’s instructions and previous studies conducted with this same species^29,46^. Intra-assay variation was 3.52% for CORT and 4.00% for T. Inter-assay variation was 3.00% for CORT and 4.06% for T. Sensitivity of the assays was 94 pg/ml and 9 pg/ml for CORT and T, respectively.

### Statistical analyses

Descriptive statistics were completed for all variables of *R. icterica* toads, and data were then submitted to Shapiro-Wilk normality test. With the exception of snout-vent length and phagocytosis percentage, all variables showed absence of normality and were transformed to fit the prerequisites of parametric tests as follows: body mass to log_10_; CORT and T to square root; bacterial killing ability and phagocytosis efficiency to arccosine. A measure of body condition (body index) was calculated as the residuals from the regression of body mass as a function of snout-vent length (original values) and included in the analysis. Additionally, presence or absence of calling behavior at the moment of capture was also included as a factor in the analysis. Pearson correlation tests were used to investigate correlations between variables in the field and throughout captivity. Data were pooled for correlation analyses during captivity (7, 30, 60, and 90 days in captivity). Since three individuals, which were not calling in field, died of unknown causes in captivity, they were not included in the analysis.

A set of ANCOVA for independent measures was used to investigate the effect of time in captivity on studied variables. CORT, T, bacterial killing ability, phagocytosis percentage and phagocytosis efficiency were used as dependent variables, body mass as a co-variable, and captivity duration as a factor. For the dependent variables not significantly affected by body mass, two sets of independent measures ANOVAs were then performed. The first included calling behavior (calling and non-calling) and captivity duration as factors. For variables not affected by calling behavior, ANOVAs were performed using only captivity duration as a factor. All ANOVAs were followed by tests for mean multiple comparisons with Bonferroni adjustment.

In order to investigate the relations among studied variables for *R. icterica* we performed structural equation modeling (SEM). Eight models were proposed based on Pearson correlation tests, with predictions based on the available knowledge about the relations between the studied physiological traits (For detailed information see: SEM description and based relations between the studied physiological traits in supplementary material). All proposed models and detailed coefficient analyses are available as supplementary material (Figs S1 and S2 and Table S7). The overall model fit was assessed based on χ^2^ statistic^76^. A nonsignificant χ^2^ result for a test (*P* > 0.05) indicates that data support the proposed model^76^. Akaike’s information criterion (AIC) was used to identify the best model among those proposed. In this way, models that were supported by χ^2^ test and had smaller AIC (dAICc < 2.0 on model selection analysis) were selected for better explaining the relationships among the variables^76^ (Table S6).

We also investigated the relationships among steroids, body condition and immunity in response to captivity maintenance in *R. schneideri* by comparing the relationships between the same studied variables for *R. icterica* in the SEM. The study conducted with *R. schneideri* was performed in October the same year (2015), including males collected in the beginning of its reproductive season^8^. The SEM for *R. schneideri* was performed by using CORT, T, phagocytosis efficiency and bacterial killing ability obtained from Titon *et al*.^8^, and included an additional variable, body index (Table S8), according to the models described for *R. icterica*. The eight proposed models for *R. schneideri* are available as supplementary material (Figs S3, S4), with the models selected for better explaining the relationships among the variables showed in Table S6. By including data from *R. schneideri*^8^ in this analysis, we intended to increase the scope of functional inter-relations between physiological variables studied. We do not mean to infer interpretations about evolutionary history and adaptation patterns in these toads^70^.

We performed descriptive statistics, correlations, ANCOVAs and ANOVAs using IBM SPSS Statistics 22. Structural equation modeling was performed in R 3.2.5 (R Development Core Team, 2016), according Titon *et al*.^8^.

## Electronic supplementary material

Supplementary materials

## Data Availability

All data are included in the supplementary materials as Tables S9 and S10.
