# Supplementary material for: Interplay among steroids, body condition and immunity in response to long-term captivity in toads
Source: Sci Rep. 2018 Nov 21;8:17168. doi: 10.1038/s41598-018-35495-0 (PMC6249311; doi:10.1038/s41598-018-35495-0)
Supplement: Supplementary file 1 — Supplementary materials [file 41598_2018_35495_MOESM1_ESM.pdf]

Supplementary Material for

Interplay among steroids, body condition and immunity in response to long-term captivity in toads

Stefanny Christie Monteiro Titon*, Braz Titon Junior, Vania Regina Assis, Gabriela Sarti Kinker, Pedro Augusto Carlos Magno Fernandes, Fernando Ribeiro Gomes

*Corresponding author e-mail: stefannychristie@gmail.com

This file includes:

SEM description and based relations between the studied physiological traits

Tables S1 to S8

Figures S1 to S4

Supplementary material references

Data Availability Tables S9 and S10

SEM description and based relations between the studied physiological traits

The structural equation modeling (SEM) is a multivariate statistical analysis used to test hypothesized cause-effect relationships. Models are proposed and tested against empirical data, and the magnitude of causal relationships between variables can be described (Hershberger et al., 2003; Shipley, 2000). When necessary, the variables should be transformed according to Box and Cox (1964) in order to present multivariate normality (Shipley, 2000) and fit the prerequisites of SEM analysis. For this study, eight models were proposed based on Pearson correlation tests, with predictions based on the available knowledge about the relations between the studied physiological traits (see below) using the "sem" function (Rosseel, 2018). The overall model fit was assessed based on $\chi 2$ statistic, which is computed comparing the observed and expected elements of the covariance matrix (Shipley, 2000). A nonsignificant result on $\chi 2$ test ($P > 0.05$) indicates that data support the proposed model (Shipley, 2000). Akaike's information criterion (AIC) was used to identify the best model among those proposed. In this way, models that were supported by $\chi 2$ test and had smaller AIC (dAICc < 2.0 on model selection analysis) were selected for better explaining the relationships among the variables (Shipley, 2000; Mazerolle, 2006).

Tested models in the present study were based on the following evidences from the literature: Glucocorticoids show catabolic effects, promoting lipid oxidation and muscle proteolysis in stress conditions (Malisch et al., 2007). Moreover, corticosterone plasma levels (CORT - the main glucocorticoid in amphibians) may be negatively related body condition (Moore et al., 2000; Janin et al., 2011). Testosterone plasma levels (T) decreases in response to stressors, being inversely associated with CORT in some studies (Paolucci et al., 1990; Narayan et al., 2012). In fact, the activation of the hypothalamus-pituitary-adrenal/interrenal axis can result in a decrease in T as a result of the inhibition of the hypothalamus-pituitary-gonad axis (Sapolsky 2002; Barsotti et al., 2017). Moreover, body index may influence CORT, with individuals displaying a better body condition showing better ability to modulate release and clearance of corticosterone (Heath & Dufty Jr., 1998; Raja-aho et al., 2010). A better body condition may be also positively associated with T (Ligon et al.,

1990; Lind & Beaupre, 2015; Madelaire and Gomes, 2016), but T exogenous administration may decrease body condition in some situations (Sockman and Schwabl, 2001; Roberts et al., 2007). Body condition and energetic resources can be directly and indirectly associated with immune response (Demas & Sakaria, 2005; Smith et al., 2007; Ruiz et al., 2010; Desprat et al., 2015). Additionally, immune cells are subjected to direct modulation by CORT and T, since they express glucocorticoid and androgen receptors (Nava-Castro et al., 2012; Segner et al., 2017).

Table S1. Descriptive statistics of body measures and plasma steroid levels for *R. icterica* male toads in field and captivity.

Variable	Group	CD	N	Mean ± SE	Min	Max
**Snout-vent length (mm)**	Calling	Field	6	111.57 ± 3.98	101.34	127.80
		7	4	121.67 ± 9.25	106.26	147.79
		30	4	104.97 ± 5.80	90.38	112.64
		60	4	109.36 ± 2.04	103.71	113.18
		90	5	103.36 ± 2.27	99.48	111.90
	Non-Calling	Field	4	114.76 ± 9.91	94.44	137.90
		7	2	97.88 ± 6.76	91.13	104.64
		30	2	127.70 ± 12.45	115.25	140.15
		60	3	107.56 ± 9.90	93.91	126.82
		90	1	91.71 ± 0.00		
**Body mass (g)**	Calling	Field	6	105.18 ± 15.44	70.10	173.70
		7	4	157.88 ± 49.78	90.01	302.26
		30	4	87.08 ± 9.99	65.96	104.89
		60	4	100.47 ± 5.96	86.01	114.59
		90	5	87.23 ± 4.39	74.71	98.57
	Non-Calling	Field	4	129.52 ± 28.60	65.80	197.40
		7	2	72.06 ± 19.66	52.40	91.72
		30	2	154.90 ± 60.83	94.08	215.73
		60	3	93.66 ± 20.90	63.86	133.95
		90	1	71.76 ± 0.00		
**Plasma corticosterone levels (ng/ml)**	Calling	Field	6	18.84 ± 4.36	9.16	34.96
		7	4	19.10 ± 6.74	10.10	38.71
		30	3	13.15 ± 3.61	8.95	20.34
		60	3	0.33 ± 0.10	0.15	0.48
		90	5	2.15 ± 0.46	1.25	3.40
	Non-Calling	Field	4	0.80 ± 0.46	0.03	1.92
		7	2	5.14 ± 0.07	5.07	5.22
		30	2	10.34 ± 2.26	8.08	12.60
		60	3	7.66 ± 0.57	7.01	8.79
		90	1	1.54 ± 0.00		
**Plasma testosterone levels (ng/ml)**	Calling	Field	5	121.16 ± 28.82	57.04	201.23
		7	4	8.64 ± 4.41	1.08	21.31
		30	4	1.74 ± 0.58	0.72	2.98
		60	3	2.54 ± 0.96	0.63	3.64
		90	5	2.36 ± 0.58	0.42	3.63
	Non-Calling	Field	3	43.41 ± 10.85	23.75	61.18
		7	2	1.87 ± 1.07	0.81	2.94
		30	2	3.00 ± 1.67	1.34	4.67
		60	3	1.39 ± 0.50	0.61	2.31
		90	1	0.40 ± 0.00		

Abbreviation as follow: **CD:** Captivity duration; **SE:** standard error; **Min:** minimum value; **Max:** maximum value.

Table S2. Descriptive statistics of immune response for *R. icterica* male toads in field and captivity.

Variable	Group	CD	N	Mean ± SE	Min	Max
**Bacterial killing ability (%)**	Calling	Field	6	93.00 ± 3.89	74	100
		7	4	98.50 ± 0.96	96	100
		30	4	100.00 ± 0.00	100	100
		60	3	62.00 ± 15.52	31	79
		90	5	64.80 ± 5.54	49	80
	Non-Calling	Field	3	97.33 ± 1.76	94	100
		7	2	92.50 ± 7.50	85	100
		30	2	96.50 ± 3.50	93	100
		60	3	74.33 ± 13.72	47	90
		90	1	69.00 ± 0.00		
**Phagocytosis (%)**	Calling	7	4	29.27 ± 6.33	17.20	40.60
		30	4	11.77 ± 2.57	7.69	19.20
		60	3	20.30 ± 6.05	13.90	32.40
		90	5	16.65 ± 4.08	5.51	24.80
	Non-calling	7	2	19.75 ± 1.65	18.10	21.40
		30	2	8.08 ± 2.32	5.77	10.40
		60	3	17.70 ± 4.80	12.60	27.30
		90	1	7.46 ± 0.00		
**Phagocytosis efficiency (%)**	Calling	7	4	10.57 ± 1.77	5.69	14.10
		30	4	5.49 ± 1.07	3.03	8.25
		60	3	10.47 ± 3.02	7.42	16.50
		90	5	8.19 ± 3.16	1.63	17.10
	Non-calling	7	2	8.95 ± 5.34	3.62	14.29
		30	2	4.01 ± 0.16	3.85	4.17
		60	3	9.90 ± 3.65	5.91	17.20
		90	1	3.38 ± 0.00		

Abbreviation as follow: **CD:** Captivity duration; **SE:** standard error; **Min:** minimum value; **Max:** maximum value.

Table S3. Effect of captivity duration on plasma steroid levels of *R. icterica* tested through a set of ANCOVAs, with plasma corticosterone and testosterone levels as dependent variables, body mass as co-variable and captivity duration (0, 7, 30, 60, and 90 days) and calling behavior (calling and non-calling) as factors.

Dependent Variable	Source	Type III SS	DF	MS	*F*	*P*
**Plasma corticosterone levels**	**Corrected Model**	137.388	18	7.633	3.994	**0.007**
	**Intercept**	0.483	1	0.483	0.253	0.623
	**Calling behavior**	0.004	1	0.004	0.002	0.963
	**CD**	6.200	4	1.550	0.811	0.540
	**BM**	0.002	1	0.002	0.001	0.978
	**Calling behavior * CD**	3.136	3	1.045	0.547	0.659
	**Calling behavior * BM**	0.022	1	0.022	0.011	0.917
	**CD * BM**	6.354	4	1.589	0.831	0.529
	**Calling behavior * CD * BM**	2.571	3	0.857	0.448	0.723
	**Error**	24.843	13	1.911		
	**Total**	577.659	32			
	**Corrected Total**	162.231	31			
**Plasma testosterone levels**	**Corrected Model**	387.857	18	21.548	6.441	**0.001**
	**Intercept**	0.000	1	0.000	0.000	0.994
	**Calling behavior**	0.829	1	0.829	0.248	0.627
	**CD**	1.894	4	0.473	0.142	0.964
	**BM**	0.392	1	0.392	0.117	0.738
	**Calling behavior * CD**	4.861	3	1.620	0.484	0.699
	**Calling behavior * BM**	0.523	1	0.523	0.156	0.699
	**CD * BM**	0.938	4	0.235	0.070	0.990
	**Calling behavior * CD * BM**	3.988	3	1.329	0.397	0.757
	**Error**	43.488	13	3.345		
	**Total**	811.290	32			
	**Corrected Total**	431.345	31			

Abbreviation as follow: **Type III SS:** Type III sum of squares; **DF:** Degrees of freedom; **MS:** Mean square; **CD:** Captivity duration; **BM:** Body mass. Variables with *P* significant < 0.05 are highlighted in bold.

Table S4. Effect of captivity duration on immune response of *R. icterica* tested through a set of ANCOVAs, with bacterial killing ability, phagocytosis percentage and phagocytosis efficiency as dependent variables, body mass as co-variable and captivity duration (0, 7, 30, 60, and 90 days) and calling behavior (calling and non-calling) as factors.

Dependent Variable	Source	Type III SS	DF	MS	*F*	*P*
Bacterial killing ability	**Corrected Model**	11416.548	18	634.253	4.302	**0.003**
	**Intercept**	2.121	1	2.121	0.014	0.906
	**Calling behavior**	19.985	1	19.985	0.136	0.718
	**CD**	1579.001	4	394.750	2.678	0.072
	**BM**	213.569	1	213.569	1.449	0.247
	**Calling behavior * CD**	872.073	3	290.691	1.972	0.162
	**Calling behavior * BM**	15.329	1	15.329	0.104	0.752
	**CD * BM**	1386.165	4	346.541	2.351	0.101
	**Calling behavior * CD * BM**	770.137	3	256.712	1.741	0.202
	**Error**	2211.388	15	147.426		
	**Total**	182957.828	34			
	**Corrected Total**	13627.936	33			
Phagocytosis	**Corrected Model**	1479.298	14	105.664	1.205	0.399
	**Intercept**	19.758	1	19.758	0.225	0.646
	**Calling behavior**	125.724	1	125.724	1.434	0.262
	**CD**	167.607	3	55.869	0.637	0.610
	**BM**	56.648	1	56.648	0.646	0.442
	**Calling behavior * CD**	207.279	2	103.639	1.182	0.350
	**Calling behavior * BM**	131.844	1	131.844	1.504	0.251
	**CD * BM**	184.649	3	61.550	0.702	0.574
	**Calling behavior * CD * BM**	214.854	2	107.427	1.225	0.338
	**Error**	789.174	9	87.686		
	**Total**	9778.938	24			
	**Corrected Total**	2268.471	23			
Phagocytosis efficiency	**Corrected Model**	408.895	14	29.207	1.548	0.258
	**Intercept**	19.154	1	19.154	1.015	0.340
	**Calling behavior**	62.944	1	62.944	3.335	0.101
	**CD**	142.230	3	47.410	2.512	0.124
	**BM**	32.942	1	32.942	1.745	0.219
	**Calling behavior * CD**	77.868	2	38.934	2.063	0.183
	**Calling behavior * BM**	67.039	1	67.039	3.552	0.092
	**CD * BM**	145.006	3	48.335	2.561	0.120
	**Calling behavior * CD * BM**	81.793	2	40.897	2.167	0.171
	**Error**	169.860	9	18.873		
	**Total**	2172.895	24			
	**Corrected Total**	578.755	23			

Abbreviation as follow: **Type III SS:** Type III sum of squares; **DF:** Degrees of freedom; **MS:** Mean square; **CD:** Captivity duration; **BM:** Body mass; Variables with *P* significant < 0.05 are highlighted in bold.

Table S5. Effect of captivity duration on immune response of *R. icterica* male toads tested through a set of ANOVAs, with bacterial killing ability, phagocytosis percentage and phagocytosis efficiency as dependent variables and captivity duration (0, 7, 30, 60, and 90 days) and calling behavior (calling and non-calling) as factors.

Dependent Variable	Source	Type III SS	DF	MS	*F*	*P*
**Bacterial killing ability**	**Corrected Model**	6182.378	9	686.931	6.827	< **0.001**
	**Intercept**	133810.573	1	133810.573	1329.809	< **0.001**
	**Calling behavior**	0.984	1	0.984	0.010	0.922
	**CD**	4394.400	4	1098.600	10.918	< **0.001**
	**Calling behavior * CD**	297.579	4	74.395	0.739	0.575
	**Error**	2314.349	23	100.624		
	**Total**	182957.828	33			
	**Corrected Total**	8496.727	32			
**Phagocytosis**	**Corrected Model**	1000.434	7	142.919	1.803	0.156
	**Intercept**	5096.587	1	5096.587	64.308	< **0.001**
	**Calling behavior**	672.997	3	224.332	2.831	0.072
	**CD**	185.651	1	185.651	2.343	0.145
	**Calling behavior * CD**	49.370	3	16.457	0.208	0.890
	**Error**	1268.038	16	79.252		
	**Total**	9778.938	24			
	**Corrected Total**	2268.471	23			
**Phagocytosis efficiency**	**Corrected Model**	135.434	7	19.348	0.698	0.673
	**Intercept**	1103.925	1	1103.925	39.842	< **0.001**
	**Calling behavior**	117.710	3	39.237	1.416	0.275
	**CD**	21.293	1	21.293	0.769	0.394
	**Calling behavior * CD**	10.005	3	3.335	0.120	0.947
	**Error**	443.321	16	27.708		
	**Total**	2172.895	24			
	**Corrected Total**	578.755	23			

Abbreviation as follow: **Type III SS:** Type III sum of squares; **DF:** Degrees of freedom; **MS:** Mean square; **CD:** Captivity duration. Variables with *P* significant < 0.05 are highlighted in bold.

Table S6. SEM analysis result including all fitted models for body index, plasma corticosterone and testosterone levels, bacterial killing ability and phagocytosis efficiency for *Rhinella icterica* and *R. schneideri*.

Species	Model	X^2	DF	$P(X^2)$	AIC	dAIC	weight
R. icterica	**Model 1***	2.841	5	0.724	610.9	0.0	0.593
	**Model 7***	2.782	4	0.595	612.9	1.9	0.225
	**Model 6**	2.506	3	0.474	614.6	3.7	0.095
	**Model 8**	7.074	5	0.215	615.2	4.2	0.071
	**Model 2**	11.086	5	0.050	619.2	8.2	0.010
	**Model 4**	9.803	3	0.020	621.9	11.0	0.003
	**Model 3**	9.803	3	0.020	621.9	11.0	0.003
	**Model 5**	10.907	3	0.012	623.0	12.1	0.001
R. schneideri	**Model 3***	1.618	3	0.655	-97.1	0.0	0.306
	**Model 4***	1.618	3	0.655	-97.1	0.0	0.306
	**Model 5***	2.243	3	0.524	-96.4	0.6	0.224
	**Model 1**	8.657	5	0.124	-94.0	3.0	0.067
	**Model 2**	8.941	5	0.111	-93.7	3.3	0.058
	**Model 7**	8.588	4	0.072	-92.1	5.0	0.026
	**Model 6**	8.873	3	0.031	-89.8	7.3	0.008
	**Model 8**	14.065	5	0.015	-88.6	8.4	0.005

Abbreviation as follow: **DF:** Degrees of freedom; **AIC:** Akaike information criterion; **dAIC:** difference between Akaike information criterion. (N = 19 and 20 for *R. icterica* and *R. schneideri*, respectively). * = Selected models ($P > 0.05$ and dAIC <2.0). The significant chi-square-P determines the model is not supported by the data. The differences between supported and non-supported models for both species were mostly related to the inter-relationships between corticosterone, body index and bacterial killing ability. In this case, the model that were not supported for *R. icterica* (models 2, 3, 4, and 5) were supported for *R. schneideri*. Meanwhile, the non-supported models for *R. schneideri* (models 6 and 8) were supported for *R. icterica*.

Table S7. SEM detailed coefficient results of each model for *Rhinella icterica.*

Model	Relation	Estimate	SE	F	*P*	Std.all
**Model 1**	BI ~ CORT	-0.062	0.537	-0.115	0.908	-0.026
	BKA ~ CORT	2.131	0.640	3.330	**0.001**	0.605
	T ~ BI	0.029	0.026	1.100	0.271	0.245
	PE ~ T	1.740	0.770	2.260	**0.024**	0.458
	BKA ~ ~ PE	6.591	14.898	0.442	0.658	0.102
**Model 2**	BI ~ CORT	-0.062	0.537	-0.115	0.908	-0.026
	T ~ BI	0.029	0.026	1.100	0.271	0.245
	BKA ~ T	0.579	2.885	0.201	0.841	0.046
	PE ~ T	1.776	0.774	2.295	**0.022**	0.466
	BKA ~ ~ PE	-5.770	18.411	-0.313	0.754	-0.072
**Model 3**	BI ~ CORT	-0.062	0.537	-0.115	0.908	-0.026
	T ~ BI	0.029	0.026	1.100	0.271	0.245
	BKA ~ BI	-0.035	0.340	-0.102	0.919	-0.023
	PE ~ BI	-0.045	0.103	-0.439	0.661	-0.100
	T ~ ~ BKA	1.229	5.291	0.232	0.816	0.053
	T ~ ~ PE	3.527	1.786	1.975	**0.048**	0.508
	BKA ~ ~ PE	-4.040	20.686	-0.195	0.845	-0.045
**Model 4**	BI ~ CORT	-0.062	0.537	-0.115	0.908	-0.026
	T ~ BI	0.029	0.026	1.100	0.271	0.245
	BKA ~ BI	-0.055	0.351	-0.156	0.876	-0.037
	BKA ~ T	0.693	2.974	0.233	0.816	0.055
	PE ~ BI	-0.102	0.091	-1.124	0.261	-0.228
	PE ~ T	1.988	0.773	2.573	**0.010**	0.522
	BKA ~ ~ PE	-6.483	17.833	-0.364	0.716	-0.084
**Model 5**	T ~ CORT	-0.017	0.063	-0.265	0.791	-0.061
	BI ~ CORT	-0.062	0.537	-0.115	0.908	-0.026
	BKA ~ BI	-0.055	0.340	-0.160	0.873	-0.037
	BKA ~ T	0.693	2.883	0.240	0.810	0.055
	PE ~ BI	-0.102	0.088	-1159	0.246	-0.222
	PE ~ T	1988	0.749	2653	**0.008**	0.507
	BKA ~ ~ PE	-6.483	17.833	-0.364	0.716	-0.084
**Model 6**	CORT ~ BI	-0.011	0.098	-0.115	0.908	-0.026
	T ~ CORT	-0.017	0.063	-0.265	0.791	-0.061
	BKA ~ T	1.036	2.320	0.446	0.655	0.082
	PE ~ T	1.724	0.750	2.298	**0.022**	0.452
	BKA ~ CORT	2.076	0.641	3.240	**0.001**	0.597
	PE ~ CORT	-0.234	0.207	-1.129	0.259	-0.222
	BKA ~ ~ PE	6.207	14.338	0.433	0.665	0.100
**Model 7**	BKA ~ CORT	2.131	0.640	3.330	**0.001**	0.605
	T ~ CORT	-0.015	0.061	-0.244	0.807	-0.054
	T ~ BI	0.029	0.026	1.095	0.273	0.243
	PE ~ T	1.740	0.770	2.260	**0.024**	0.458
	BI ~ CORT	-0.062	0.537	-0.115	0.908	-0.026
	BKA ~ ~ PE	6.591	14.898	0.442	0.658	0.102
**Model 8**	BKA ~ CORT	2.158	0.637	3.388	**0.001**	0.610
	T ~ CORT	-0.017	0.063	-0.265	0.791	-0.061
	BI ~ T	2.076	1.886	1.100	0.271	0.245
	PE ~ BI	-0.045	0.102	-0.438	0.661	-0.099
	BKA ~ ~ PE	10.148	16.831	0.603	0.547	0.140

Abbreviation as follow: **BI:** Body index; **CORT:** plasma corticosterone levels; **BKA:** bacterial killing ability; **T:** plasma testosterone levels; **PE:** phagocytosis efficiency; **" ~ "** Represents a regression result; **" ~ ~ "** represents a correlation result; **Estimate:** Estimate coefficient; **SE:** Standard error; **Std. all:** Completely standardized solution. (*N* = 19 and 20 for *R. icterica* and *R. schneideri*, respectively).

Table S8. SEM detailed coefficient results of each model for *Rhinella schneideri*.

Model	Relation	Estimate	SE	F	*P*	Std.all
**Model 1**	BI ~ CORT	-0.607	0.277	-2.192	**0.028**	-0.607
	BKA ~ CORT	-0.245	0.436	-0.561	0.575	-0.245
	T ~ BI	0.085	0.080	1.062	0.288	0.085
	PE ~ T	0.328	0.222	1.480	0.139	0.328
	BKA ~ ~ PE	0.003	0.005	0.581	0.561	0.003
**Model 2**	BI ~ CORT	-0.607	0.277	-2.192	**0.028**	-0.607
	T ~ BI	0.085	0.080	1.062	0.288	0.085
	BKA ~ T	-0.133	0.876	-0.151	0.880	-0.133
	PE ~ T	0.321	0.223	1.436	0.151	0.321
	BKA ~ ~ PE	0.003	0.005	0.644	0.519	0.003
**Model 3**	BI ~ CORT	-0.607	0.277	-2.192	**0.028**	-0.607
	T ~ BI	0.085	0.080	1.062	0.288	0.085
	BKA ~ BI	0.680	0.284	2.393	**0.017**	0.680
	PE ~ BI	-0.036	0.086	-0.420	0.675	-0.036
	T ~ ~ BKA	-0.003	0.005	-0.735	0.462	-0.003
	T ~ ~ PE	0.002	0.001	1.432	0.152	0.002
	BKA ~ ~ PE	0.004	0.005	0.861	0.389	0.004
**Model 4**	BI ~ CORT	-0.607	0.277	-2.192	**0.028**	-0.607
	T ~ BI	0.085	0.080	1.062	0.288	0.085
	BKA ~ BI	0.730	0.288	2.536	**0.011**	0.730
	BKA ~ T	-0.592	0.783	-0.755	0.450	-0.592
	PE ~ BI	-0.067	0.083	-0.805	0.421	-0.067
	PE ~ T	0.363	0.226	1.606	0.108	0.363
	BKA ~ ~ PE	0.005	0.005	1.174	0.240	0.005
**Model 5**	T ~ CORT	-0.077	0.112	-0.692	0.489	-0.077
	BI ~ CORT	-0.607	0.277	-2.192	**0.028**	-0.607
	BKA ~ BI	0.730	0.281	2.600	**0.009**	0.730
	BKA ~ T	-0.592	0.764	-0.775	0.439	-0.592
	PE ~ BI	-0.067	0.081	-0.825	0.409	-0.067
	PE ~ T	0.363	0.220	1.647	0.100	0.363
	BKA ~ ~ PE	0.005	0.005	1.174	0.240	0.005
**Model 6**	CORT ~ BI	-0.319	0.146	-2.192	**0.028**	-0.319
	T ~ CORT	-0.077	0.112	-0.692	0.489	-0.077
	BKA ~ T	-0.222	0.877	-0.253	0.800	-0.222
	PE ~ T	0.301	0.224	1.341	0.180	0.301
	BKA ~ CORT	-0.295	0.444	-0.665	0.506	-0.295
	PE ~ CORT	-0.068	0.114	-0.595	0.552	-0.068
	BKA ~ ~ PE	0.003	0.005	0.571	0.568	0.003
**Model 7**	BKA ~ CORT	-0.245	0.436	-0.561	0.575	-0.245
	T ~ CORT	-0.032	0.123	-0.262	0.793	-0.032
	T ~ BI	0.075	0.089	0.840	0.401	0.075
	PE ~ T	0.328	0.222	1.480	0.139	0.328
	BI ~ CORT	-0.607	0.277	-2.192	**0.028**	-0.607
	BKA ~ ~ PE	0.003	0.005	0.581	0.561	0.003
**Model 8**	BKA ~ CORT	-0.185	0.432	-0.429	0.668	-0.185
	T ~ CORT	-0.077	0.112	-0.692	0.489	-0.077
	BI ~ T	0.629	0.592	1.062	0.288	0.629
	PE ~ BI	-0.068	0.085	-0.802	0.423	-0.068
	BKA ~ ~ PE	0.005	0.006	0.834	0.404	0.005

Abbreviation as follow: **BI:** Body index; **CORT:** plasma corticosterone levels; **BKA:** bacterial killing ability; **T:** plasma testosterone levels; **PE:** phagocytosis efficiency; **" ~ "** Represents a regression result; **" ~ ~ "** represents a correlation result; **Estimate:** Estimate coefficient; **SE:** Standard error; **Std. all:** Completely standardized solution. (*N* = 19 and 20 for *R. icterica* and *R. schneideri*, respectively).

Figure S1

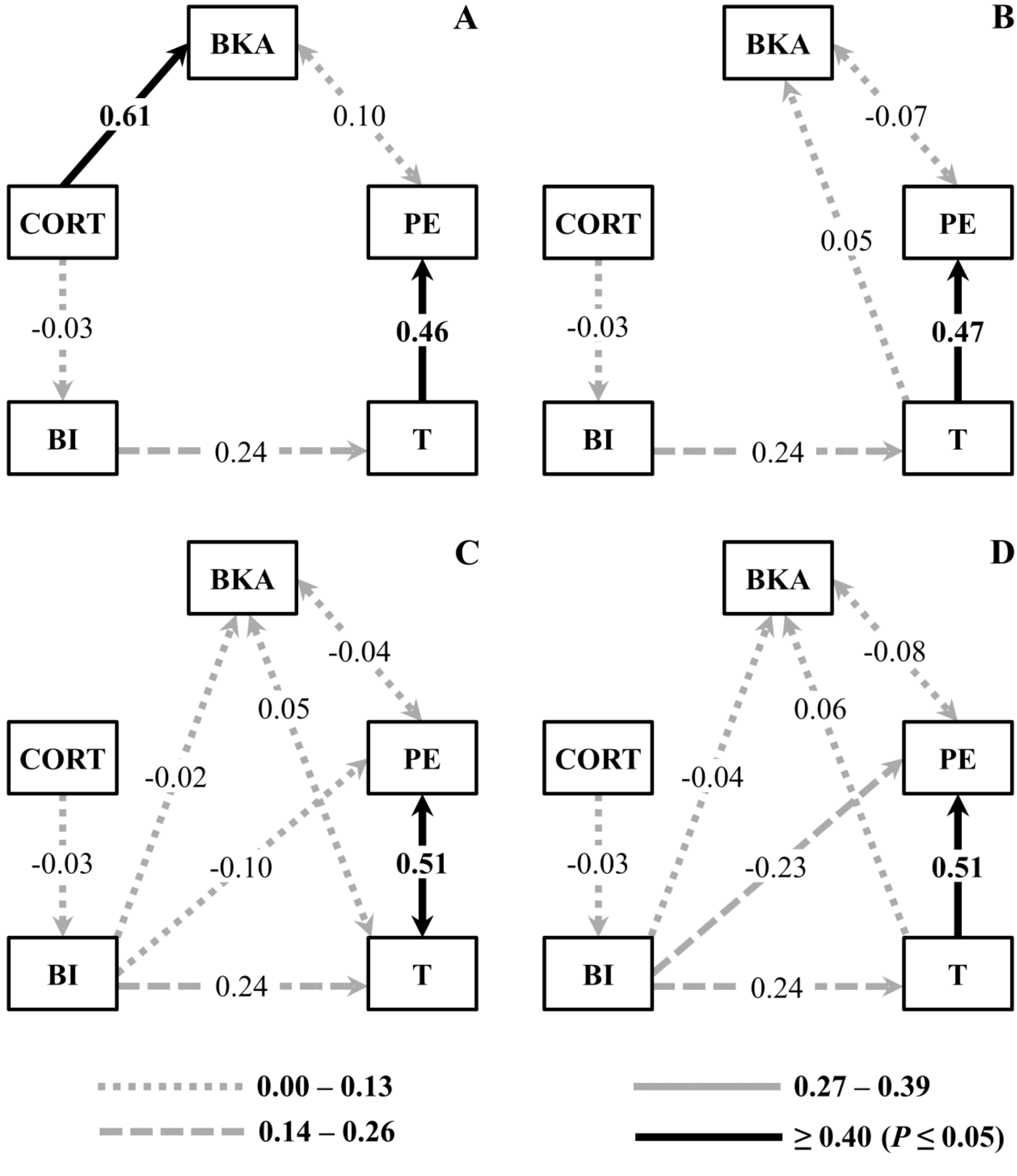

Figure S1. Proposed models (1-4) for *R. icterica*. Path diagrams of four causal models tested for *R. icterica*. Path coefficients shown are all standardized values (A) Model 1; (B) Model 2; (C) Model 3; (D) Model 4. Numbers within arrow means the completely standardized solution coefficient values. Positive numbers represent positive relations and negative numbers represent negative relations. One-arrow represents a regression result and two-arrow represent a correlation result. Abbreviation as follow: **BKA:** Bacterial killing ability; **CORT:** Plasma corticosterone levels; **BI:** Body index; **PE:** Phagocytosis efficiency; **T:** Plasma testosterone levels. (N = 19).

Figure S2

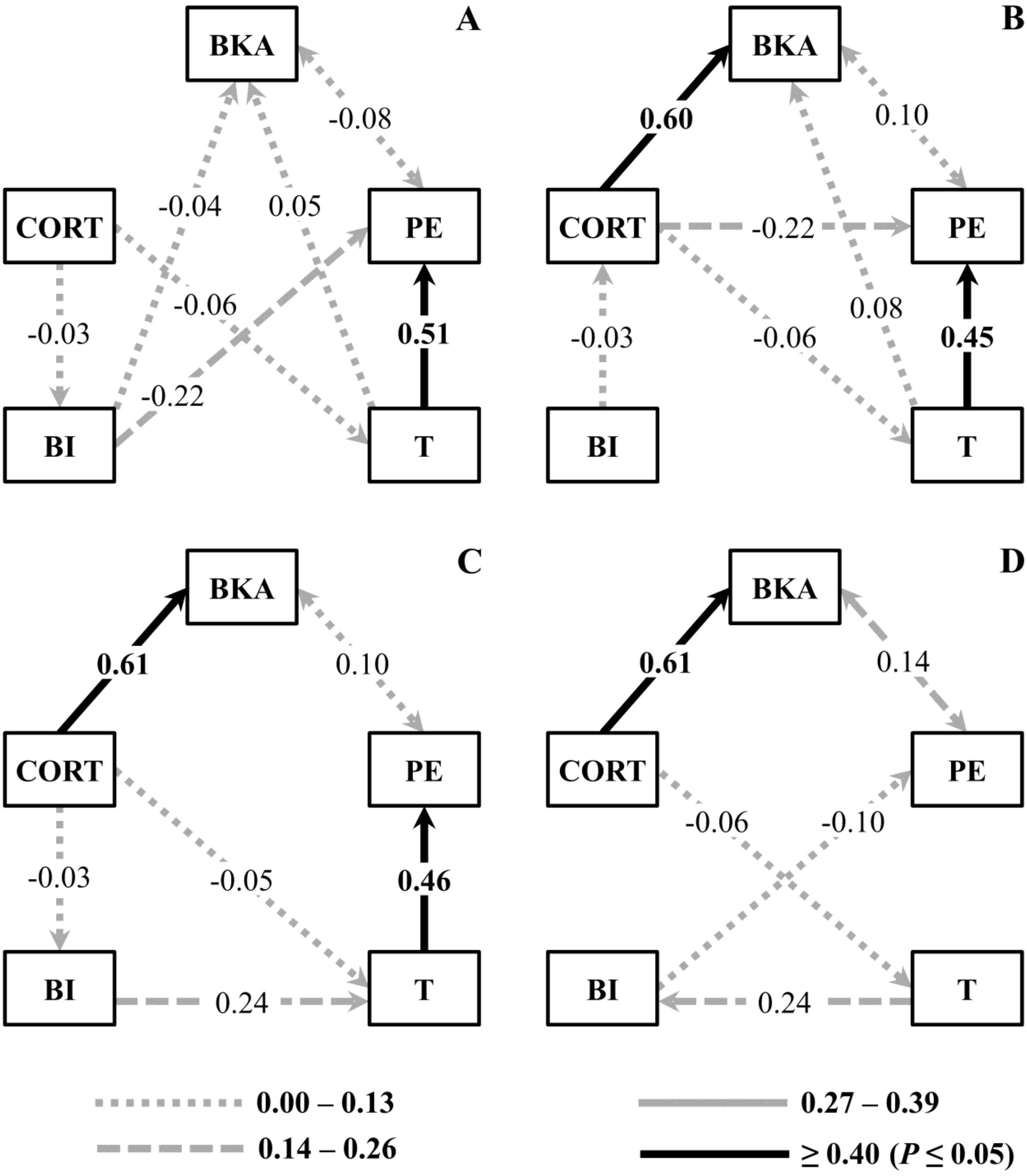

Figure S2. Proposed models (5-8) for *R. icterica*. Path diagrams of four causal models tested for *R. icterica*. Path coefficients shown are all standardized values (A) Model 5; (B) Model 6; (C) Model 7; (D) Model 8. Numbers within arrow means the completely standardized solution coefficient values. Positive numbers represent positive relations and negative numbers represent negative relations. One-arrow represents a regression result and two-arrow represent a correlation result. Abbreviation as follow: **BKA:** Bacterial killing ability; **CORT:** Plasma corticosterone levels; **BI:** Body index; **PE:** Phagocytosis efficiency; **T:** Plasma testosterone levels. (N = 19).

Figure S3

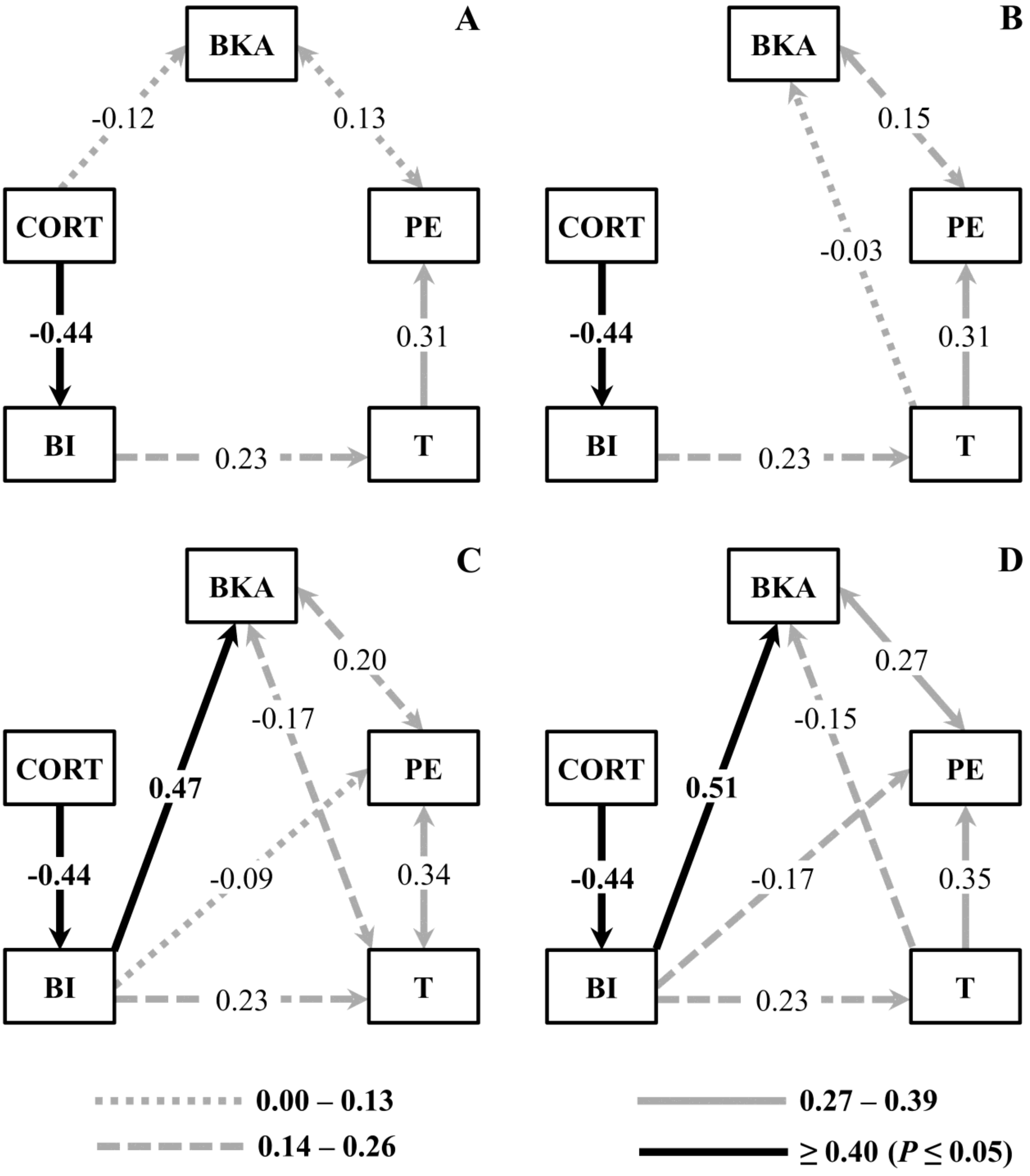

Figure S3. Proposed models (1-4) for *R. schneideri*. Path diagrams of four causal models tested for *R. schneideri*. Path coefficients shown are all standardized values (A) Model 1; (B) Model 2; (C) Model 3; (D) Model 4. Numbers within arrow means the completely standardized solution coefficient values. Positive numbers represent positive relations and negative numbers represent negative relations. One-arrow represents a regression result and two-arrow represent a correlation result. Abbreviation as follow: **BKA:** Bacterial killing ability; **CORT:** Plasma corticosterone levels; **BI:** Body index; **PE:** Phagocytosis efficiency; **T:** Plasma testosterone levels. (N = 20).

Figure S4

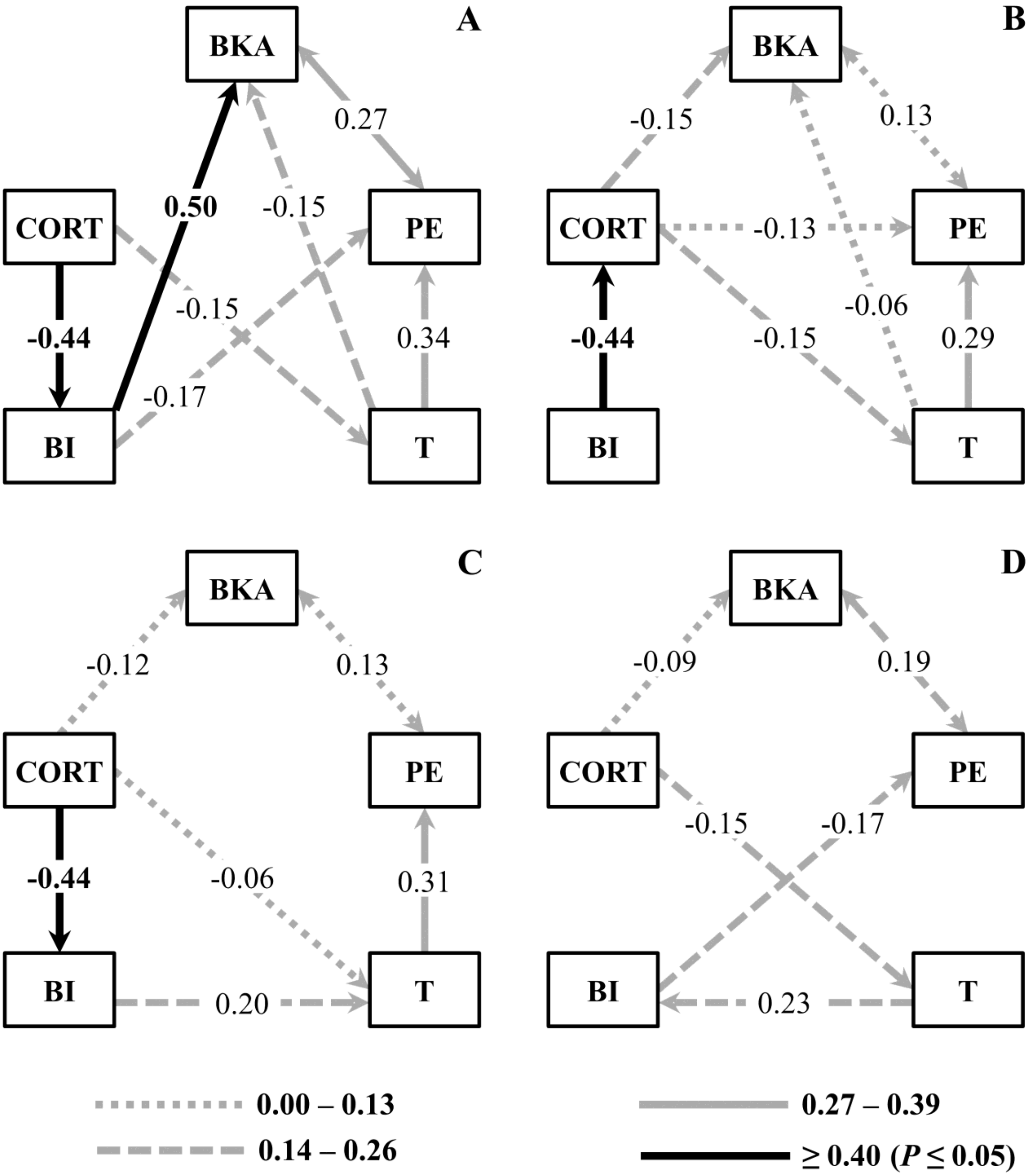

Figure S4. Proposed models (5-8) for *R. schneideri*. Path diagrams of four causal models tested for *R. schneideri*. Path coefficients shown are all standardized values (A) Model 5; (B) Model 6; (C) Model 7; (D) Model 8. Numbers within arrow means the completely standardized solution coefficient values. Positive numbers represent positive relations and negative numbers represent negative relations. One-arrow represents a regression result and two-arrow represent a correlation result. Abbreviation as follow: **BKA:** Bacterial killing ability; **CORT:** Plasma corticosterone levels; **BI:** Body index; **PE:** Phagocytosis efficiency; **T:** Plasma testosterone levels. (N = 20).

Supplementary material references

Barsotti, A.M.G., Assis, V.R., Titon, S.C.M., Titon Jr., Ferreira, Z.F.S., Gomes, F.R., 2017. ACTH modulation on corticosterone, melatonin, testosterone and innate immune response in the treefrog *Hypsiboas faber*. Comp. Biochem. Physiol. A 204: 177-184.

Box, G.E.P., Cox, D.R. (1964). An analysis of transformations. J. Roy. Stat. Soc. B Met. 26(2): 211–252.

Demas, G.E., Sakaria, S. (2005). Leptin regulates energetic tradeoffs between body fat and humoural immunity. Proc. R. Soc. B 272: 1845-1850.

Heath, J.A., Dufty Jr., A.M. (1998). Body condition and the adrenal stress response in captive American kestrel juveniles. Physiol. Zool. 71(1): 67-73.

Hershberger, S.L., Marcoulides, G.A., Parramore, M.M. (2003). Structural equation modeling: An introduction. *In* B. H. Pugesek, A.A. Eye, Tomer, A. (Eds.), Structural equation modeling: Applications in ecological and evolutionary biology. London, England: Cambridge University Press. Pp: 3-41.

Janin, A., Léna, J.P., Joly, P. (2011). Beyond occurrence: Body condition and stress hormone as integrative indicators of habitat availability and fragmentation in the common toad. Biol. Conserv. 144: 1008-1016.

Ligon, J.D., Thornhill, R., Zuk, M., Johnson, K. (1990). Male-male competition, ornamentation and the role of testosterone in sexual selection in red jungle fowl. Anim. Behav. 40: 367-373.

Lind, C.M., Beaupre, S.J. (2015). Male snakes allocate time and energy according to individual energetic status: body condition, steroid hormones, and reproductive behavior in timber rattlesnakes (*Crotalus horridus*). Physiol. Biochem. Physiol. 88(6): 624-633.

Madelaire, C.B., Gomes, F.R. (2016). Breeding under unpredictable conditions: Annual variation in gonadal maturation, energetic reserves and plasma levels of androgens and corticosterone in anurans from the Brazilian semi-arid. Gen. Comp. Endocrinol. 228: 9-16.

Malisch, J.L., Saltzman, W., Gomes, F.R., Rezende, E.L., Jeske, D.R., Garland Jr., T. (2007). Baseline and stress-induced plasma corticosterone concentrations of mice selectively bred for high voluntary wheel running. Physiol. Biochem. Zool. 80: 146–156.

Mazerolle, M. (2006). Improving data analysis in herpetology: Using Akaike's Information Criterion (AIC) to assess the strength of biological hypotheses. Amphibia-Reptilia. 27(2): 169–180.

Moore, I.T., Lerner, J.P., Lerner, D.T., Mason, R.T. (2000). Relationships between annual cycles of testosterone, corticosterone, and body condition in male red-spotted garter snakes, *Thamnophis sirtalis concinnus*. Physiol. Biochem. Zool. 73(3): 307-312.

Nava-Castro, K., Hernández-Bello, R., Muñiz-Hernandez, S., Camacho-Arroyo, I., Morales-Montor, J. (2012). Sex steroids, immune system, and parasitic infections: facts and hypotheses. An. N Y Acad. Sci. 1262: 16-26.

Narayan, E.J., Hero, J., Cockrem, J.F. (2012). Inverse urinary corticosterone and testosterone metabolite responses to different durations of restraint in the cane toad (*Rhinella marina*). Gen. Comp. Endocrinol. 179: 345-349.

Paolucci, M., Esposito, V., Di Fiore, M.M., Botte, V. (1990). Effects of short postcapture confinement on plasma reproductive hormone and corticosterone profiles in *Rana esculenta* during the sexual cycle. Boll. Zool. 57: 253-259.

Raja-Aho, S., Suorsa, P., Vainio, M., Nikimmara, M., Lehikoinen, E., Eeva, T. (2010). Body condition is associated with adrenocortical response in the barn swallow (Hirundo rustica L.) during early stages of autumn migration. Oecologia. DOI: 10.1007/s00442-009-1553-0.

Roberts, M.L., Buchanan, K.L., Hasselquist, D., Evans, M.R. (2007). Effects of testosterone and corticosterone on immunocompetence in the zebra finch. Horm. Behav. 51: 126-134.

Rosseel, Y. (2018). The lavaan tutorial. Link: http://lavaan.ugent.be/tutorial/tutorial.pdf (Accessed in: 09/05/2018).

Ruiz, M., French, S.S., Demas, G.E., Martins, E.P. (2010). Food supplementation and testosterone Interact to influence reproductive behavior and immune function in *Sceloporus graciosus*. Horm. Behav. 57: 134-139.

Sapolsky, R.M. (2002). Endocrinology of the stress response. In: Becker, J. B., Reedlove, S. M., Crews, D., McCarthy, M. M. (eds.), Behavioral Endocrinology. Cambridge: MIT press. Pp 409-450.

Segner, H., Kemenade, B.M.L.V., Chadzinska, M. (2017). The immunomodulatory role of the hypothalamus-pituitary-gonad axis: proximate mechanism for reproduction-immune trade-offs? Dev. Comp. Immunol. 66: 43-60.

Shipley, B. (2000). Cause and correlation in biology. Cambridge, England: Cambridge University Press.

Smith, H.G., Raberg, L., Ohlsson, T., Granbom, M., Hasselquist, D. (2007). Carotenoid and protein supplementation have differential effects on pheasant ornamentation and immunity. J. Evolution. Biol. 20(1): 310-319.

Sockman, K.W., Schwalb, H. (2001). Plasma corticosterone in nestling American kestrels: effects of age, handling stress, yolk androgens and body condition. Gen. Comp. Endocrinol. 122: 205-212.

Titon, S.C.M., Assis. V.R., Titon Jr, B., Cassettari, B.O., Fernandes, P.A.C.M., Gomes, F.R. (2017). Captivity effects on immune response and steroid plasma levels of a Brazilian toad (*Rhinella schneideri*). J. Exp. Zool. 327(2-3): 127-138.

Data Availability Table S9. Full data of *Rhinella icterica* toads.

Call Behav	Day	CORT	T	Body mass	SVL	BKA	PP	PE
No	0	1.92	23.75	65.80	94.44	-	-	-
No	0	0.04	-	197.40	137.90	100	-	-
No	0	0.05	45.29	151.30	123.98	98	-	-
No	0	1.21	61.18	103.60	102.71	94	-	-
Yes	0	11.40	113.30	173.70	127.80	100	-	-
Yes	0	9.16	62.37	109.80	117.26	96	-	-
Yes	0	27.29	201.23	70.10	101.34	94	-	-
Yes	0	34.96	-	113.40	112.05	98	-	-
Yes	0	9.50	171.88	87.90	105.10	74	-	-
Yes	0	20.73	57.04	76.20	105.90	96	-	-
No	7	5.22	2.94	52.40	91.13	85	21.40	14.29
No	7	5.07	0.81	91.72	104.64	100	18.10	3.62
Yes	7	17.29	-	145.72	121.33	98	19.50	5.69
Yes	7	-	1.08	93.53	111.30	96	39.80	10.70
Yes	7	10.32	4.91	90.01	106.26	100	17.20	11.80
Yes	7	10.10	7.25	-	147.79	100	40.60	14.10
No	30	8.08	1.34	94.08	115.25	100	10.40	4.17
No	30	12.60	4.67	215.73	140.15	93	5.77	3.85
Yes	30	10.18	2.98	65.96	90.38	100	19.20	8.25
Yes	30	-	0.72	104.89	115.80	100	7.69	5.13
Yes	30	8.95	2.47	74.11	101.08	100	11.11	5.55
Yes	30	20.34	0.79	103.36	112.64	100	9.09	3.03
No	60	7.19	2.31	133.95	126.82	47	13.20	6.60
No	60	7.02	0.61	83.16	101.96	90	12.60	5.91
No	60	8.79	1.25	63.86	93.91	86	27.30	17.20
Yes	60	-	-	103.56	111.18	-	14.60	7.48
Yes	60	0.15	0.63	97.72	109.37	31	-	-
Yes	60	0.49	3.64	114.59	113.18	79	32.40	16.50
Yes	60	0.37	3.36	86.01	103.71	76	13.90	7.42
No	90	1.54	0.40	71.76	91.71	69	7.46	3.38
Yes	90	1.25	3.00	79.87	99.63	72	20.90	6.10
Yes	90	1.68	3.01	98.57	111.90	80	24.80	17.10
Yes	90	3.40	1.72	89.24	102.31	67	5.51	2.00
Yes	90	3.14	0.42	74.71	99.48	56	8.13	1.63
Yes	90	1.29	3.63	93.75	103.50	49	23.90	14.10

Abbreviation as follow: **Call behav:** presence (yes) or absence (no) of calling behaviour in the field at the moment of capture; **Day:** days in captivity, where 0 represents field data; **CORT:** plasma corticosterone levels; **T:** plasma testosterone levels; **SVL:** snout-vent-length; **BKA:** bacterial killing ability; **PP:** phagocytosis percentage; **PE:** phagocytosis efficiency; "**-**" data not available.

Data Availability Table S10. Data of *Rhinella icterica* and *R. schneideri* for SEM analyses.

Species	CORT	T	BKA	Body index	PE
Rhinella icterica	5.22	2.94	85	-2.37	14.29
	5.07	0.81	100	1.19	3.62
	10.32	4.91	100	-4.80	11.80
	8.08	1.34	100	-24.52	4.17
	12.60	4.67	93	31.23	3.85
	10.18	2.98	100	13.17	8.25
	8.95	2.47	100	-6.99	5.55
	20.34	0.79	100	-8.34	3.03
	7.19	2.31	47	-15.27	6.60
	7.02	0.61	90	-0.27	5.91
	8.79	1.25	86	1.73	17.20
	0.49	3.64	79	1.46	16.50
	0.37	3.36	76	-2.05	7.42
	1.54	0.40	69	15.45	3.38
	1.25	3.00	72	2.60	6.10
	1.68	3.01	80	-11.17	17.10
	3.40	1.72	67	4.88	2.00
	3.14	0.42	56	-2.16	1.63
	1.29	3.63	49	6.24	14.10
Rhinella schneideri*	1.27	0.14	51	4.60	1.32
	0.65	0.16	69	2.23	0.90
	6.70	0.22	67	-10.08	1.14
	2.60	0.18	69	-5.87	2.20
	6.26	0.94	62	-16.21	2.35
	4.96	0.15	56	1.74	1.18
	0.95	1.72	49	23.98	0.00
	0.38	0.25	49	-1.95	2.43
	4.01	0.34	98	18.70	1.68
	0.16	0.85	100	1.90	8.47
	1.37	0.69	96	8.89	5.01
	11.14	0.23	71	-12.95	0.00
	2.49	4.96	80	22.16	2.99
	6.59	2.60	69	0.93	2.82
	3.19	4.41	36	0.23	3.57
	3.17	1.52	56	-2.84	2.31
	8.82	0.25	48	-4.88	9.43
	2.52	1.70	36	-16.56	3.92
	4.62	0.31	9	-15.94	1.94
	6.46	0.18	67	1.93	0.76

Abbreviation as follow: **CORT:** plasma corticosterone levels; **T:** plasma testosterone levels; **BKA:** bacterial killing ability; **PE:** phagocytosis efficiency. (N = 19 and 20 for *R. icterica* and *R. schneideri*, respectively). ***** Data for CORT, T, BKA and PE for *R. schneideri* where published in Titon et al., 2017.
